# Measurement of stretch-evoked brainstem function using fMRI

**DOI:** 10.1038/s41598-021-91605-5

**Published:** 2021-06-15

**Authors:** Andrea Zonnino, Andria J. Farrens, David Ress, Fabrizio Sergi

**Affiliations:** 1 Human Robotics Laboratory, Department of Biomedical Engineering, University of Delaware, Newark, DE 19713 USA; 2 Department of Neuroscience, Baylor College of Medicine, Houston, TX 77020 USA

**Keywords:** Biomedical engineering, Cerebellum, Motor cortex, Spinal cord

## Abstract

Knowledge on the organization of motor function in the reticulospinal tract (RST) is limited by the lack of methods for measuring RST function in humans. Behavioral studies suggest the involvement of the RST in long latency responses (LLRs). LLRs, elicited by precisely controlled perturbations, can therefore act as a viable paradigm to measure motor-related RST activity using functional Magnetic Resonance Imaging (fMRI). Here we present StretchfMRI, a novel technique developed to study RST function associated with LLRs. StretchfMRI combines robotic perturbations with electromyography and fMRI to simultaneously quantify muscular and neural activity during stretch-evoked LLRs without loss of reliability. Using StretchfMRI, we established the muscle-specific organization of LLR activity in the brainstem. The observed organization is partially consistent with animal models, with activity primarily in the ipsilateral medulla for flexors and in the contralateral pons for extensors, but also includes other areas, such as the midbrain and bilateral pontomedullary contributions.

## Introduction

Multiple secondary pathways are known to participate, together with the corticospinal tract (CST), in controlling motor actions^1^. Among these secondary pathways, the reticulospinal tract (RST) is especially important for its involvement in locomotion^2^, maintenance of posture^3^, reaching^4^ and grasping^5^.

Anatomically, the RST is divided in two distinct pathways^6^: the medial tract that originates in the pons, and the lateral tract that originates in the medulla. In contrast to the CST that has a lateralized organization, with 85% of the fibers that cross the midline innervating contralateral muscles^1,7^, the RST shows a bilateral organization. A single axon that originates in either side of the reticular formation (RF) can innervate muscles on both sides of the body^4,8,9^, with stimulation of the RF that has been observed to produce bilateral muscle activity^8,10,11^. Moreover, while the CST is known to provide excitatory stimuli primarily to contralateral muscles^12^, the most widely accepted model of motor organization in the RST indicates that the medial RST provides excitation to the ipsilateral extensors and inhibition to the contralateral flexors, and the lateral RST provides inhibition to the contralateral extensors and excitation to the ipsilateral flexors^5,8,10,13^.

Although secondary to the corticospinal tract, the RST could assume considerable importance for studying the neural basis of motor impairment and recovery after corticospinal lesions. While previous research advanced that the RST could be a useful target for neuro-rehabilitative interventions that aim to strengthen neural drive to skeletal muscles^8,10,14,15^, recent research also shows that in humans increased RST function or structural integrity is associated with post-stroke impairment, including loss of independent joint control and hyperexcitability of stretch reflex^16–18^. However, given the limited capability of measuring directly and in-vivo function in the brainstem nuclei originating the RST, a complete understanding of the role of RST in neuromotor impairment and recovery is currently lacking. As such, a new technique capable of quantifying in-vivo function of the RF during motor tasks would be very useful for both basic and translational neuroscience.

Unfortunately, due to the small size of the brainstem and its location deep in the cranium, direct measurements of RF function during active motor tasks is not feasible, as the only viable approach would be using non-invasive technique based on neuroimaging. However, the between-subject and between-task variability associated with experiments involving voluntary responses, combined with the relatively low signal-to-noise ratio of functional neuroimaging of the brainstem^19^, make these investigations very challenging.

Several recent studies indicate that the RF might be actively involved in modulating the amplitude of long latency responses (LLRs), a stereotypical response evoked in muscles after a perturbation stretching a set of muscles^20^, with response times for upper extremity comprised between 50 and 100 ms. LLRs are a fundamental component of motor control, as they gracefully blend the fast reaction time afforded by short latency reflexes (SLR) (delay shorter than 50 ms) with the flexible and skilled action of voluntary movement (responses occurring more than 100 ms after stimulus). We know that the LLR follows a transcortical pathway with a prominent role of the primary motor cortex^21,22^, but the RF also processes some features of LLRs^20^. Direct evidence on the involvement of the RF in LLRs comes from neuronal recordings in behaving cats, where a burst in RF activity was detected in response to a foot drop^23^. In humans, a direct link between RF function and LLRs has not yet been established, primarily due to the lack of direct methods for measuring RF function in humans. It is currently established that LLR responses are due to the temporal overlap of two responses, the task-dependent component and the automatic response^24,25^, and it is possible that these responses may be produced by non-identical pathways. Using the StartReact paradigm, investigators have established that startle stimuli can elicit the discharge of a planned motor action of upper limb muscles within 70 ms from the stimulus, compared to the 100 ms response time measured in absence of startle^26,27^. The measured reduction in response time likely reflects the engagement of the RF in the StartReact paradigm since those circuits are directly responsible for the startle response. Moreover, the LLR task-dependent component and StartReact have been observed to share striking similarities at the level of muscle recordings and modulation by experimental factors^28^.

Because LLRs are “semi-reflexive” responses, they are less affected by confounds such as individual subject skill and task performance, resulting in smaller between-subject variability than is found for voluntary motor tasks. As such, precisely evoked LLRs may be a means to reliably stimulate the RST to enable the direct measurement of motor activity of the RF associated with rapid joint stretch using neuroimaging. Yet, while the approaches used in previous research, based on startle stimuli and/or inhibition of cortical areas, did indeed allow to conclude that brainstem regions such as the RF might be involved in LLRs, the knowledge gained via those investigations had little spatial specificity. As such, details on which areas of the RF are associated with LLRs of specific muscles are still unknown.

Here, we present StretchfMRI, a novel technique that we have developed to study the brainstem correlates of LLRs in-vivo in humans. StretchfMRI combines robotic perturbations with electromyography (EMG) and functional Magnetic Resonance Imaging (fMRI) to provide simultaneous recording of neural and muscular activity associated with LLRs. In this paper, we demonstrate that StretchfMRI enables the reliable quantification of both EMG and fMRI associated with LLRs, and establish muscle-specific representation of LLR activity in the brainstem. Additionally, we present an exploratory analysis to determine cortical areas associated with LLR activity for flexors and extensors. Some of the development stages toward StretchfMRI (including the robotic perturbator and the use of fMRI sequences with silent windows) have been presented in preliminary form in an earlier conference paper^29^. The novel components presented here for the first time include the methods for simultaneous measurement of EMG and fMRI, their validation, and a human-subject experiment to identify RF activity associated with LLRs of a flexor and an extensor muscle.

## Materials and methods

### StretchfMRI technique

**Figure 1:**
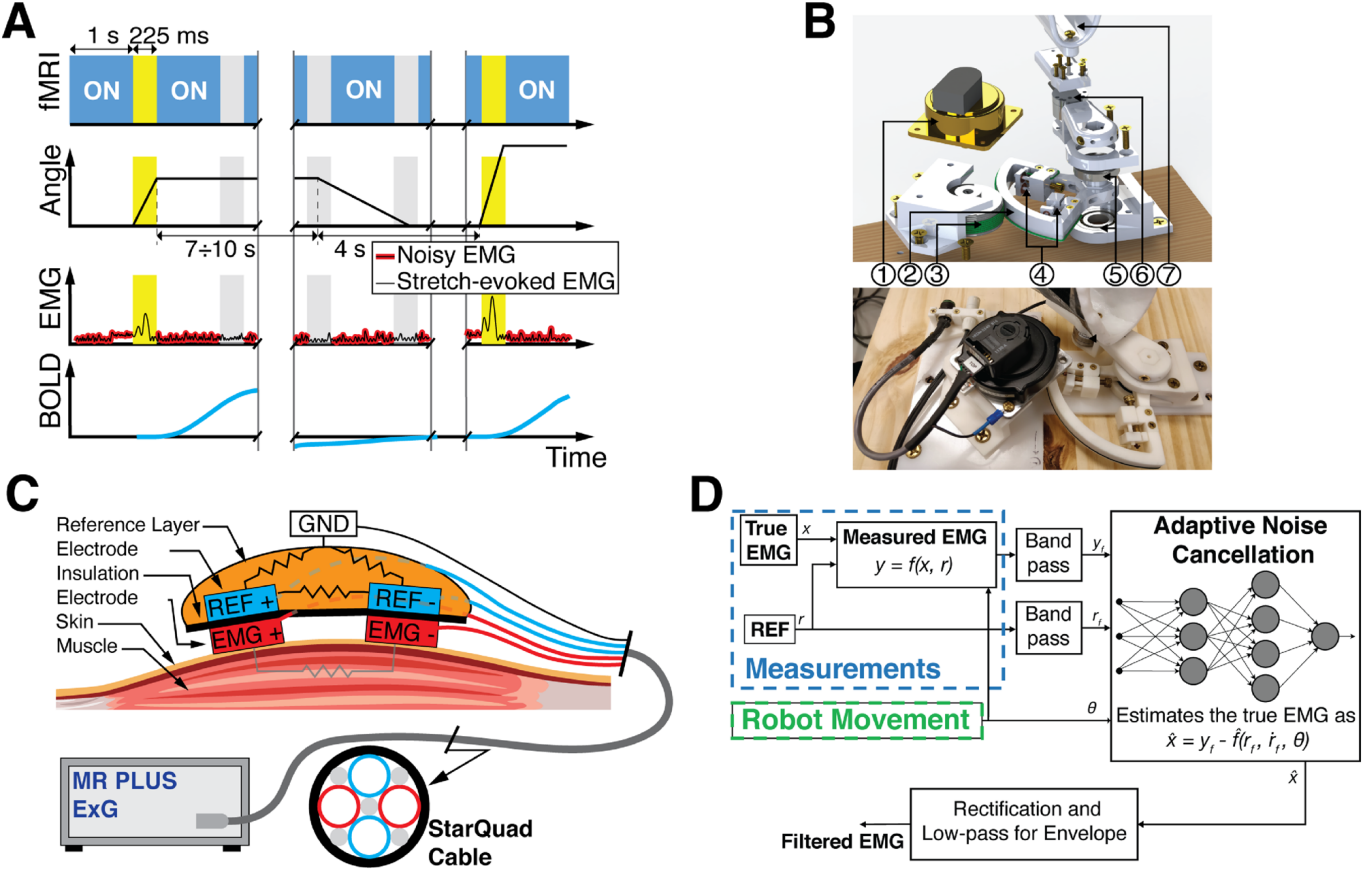
(**A**) Timing diagram of the MRI sequence (top row) with the 225 ms silent windows between acquisition of different volumes; (second row) commanded robot joint trajectory; (third row) expected EMG signal, which is clean during MRI silent windows; (bottom row) expected BOLD signal associated with the LLR; (**B**) Exploded view (top) and prototype (bottom) of the MR-StretchWrist. (1) Ultrasonic motor, (2) output capstan arc, (3) input pulley, (4) tensioning mechanisms, (5) structural bearings, (6) force/torque sensor, (7) hand support; (**C**) Hardware of the novel apparatus developed for the acquisition of the EMG data during fMRI protocols (**D**) EMG processing scheme based on Adaptive Noise Cancellation ( schemes for the other processing pipelines used for this work are reported in Fig. S7). (**A**) and (**B**) are modified from^29^.

The investigation of the neural substrates of LLRs via fMRI requires simultaneous measurement of muscular and neural activity during the application of velocity-controlled perturbations that condition LLRs in a set of muscles. To enable reliable measurement of stretch-evoked muscle responses during fMRI, we combined a newly-developed MRI-compatible robot with custom EMG acquisition and processing methods, and used a modified fMRI sequence including a 225 ms silent window after every acquisition volume, during which stretch-evoked responses were measured (Fig. 1). A one degree-of-freedom wrist robot—the MR-StretchWrist—shown in Fig. 1B and described in detail in a previous publication^29^, was used to apply perturbations at different velocities to condition LLRs of two muscles (Flexor Carpi Radialis—FCR, and Extensor Carpi Ulnaris—ECU). Muscle responses were quantified using a custom electrode set that included co-located measurement and reference electrodes. Use of the custom electrode set allowed to simultaneously record both muscle signal and motion artifact induced by the unavoidable movement of the electrodes in the scanner. The measured signals were filtered using a pipeline that included adaptive noise cancellation to estimate and remove the signal related to motion artifacts (non-linearly related to the measurement from the reference electrode), and obtain clean measurements of EMG. Details about the methods developed for this study, the protocols used for their validation, and application of the methods to study the neural substrates fo LLR are provided in the sections below.

#### Simultaneous recording of fMRI and EMG data

Measuring EMG during fMRI protocols is challenging because of the artifacts introduced in the EMG recordings by the coupling of the time- and spatially-varying electromagnetic fields required for MRI—i.e. static magnetic field, gradient magnetic field, radio waves—with the undesired movement of the EMG electrodes. While the radio waves introduce noise at a frequency range that is distinct from the spectrum expected for physiological muscle contractions, meaning that noise could be removed using frequency domain filters, this is not the case for gradient and movement artifacts whose spectrum overlaps with the one expected for muscle contractions.

Because we were only interested in measuring EMG during brief periods of time—i.e. those where we expect to observe a LLR—we introduced in the MRI scanning protocol a 225 ms silent window after each acquisition volume. During silent windows, no fMRI excitation is generated, allowing us to avoid the effects of gradient and radio-frequency waves (Fig. 1A). This approach enables the analysis of associations between EMG and fMRI signals due to the intrinsic delay of the hemodynamic signal. Such a delay decouples temporally the measurement of muscle activity associated with an LLR—expected within 100 ms of a perturbation—with the measurement of the associated blood-oxygen level dependent (BOLD) signal—which completes its course several seconds after a reflex is elicited (Fig. 1A).

While this approach allowed us to remove artifacts associated with both radio waves and gradients, preliminary analyses have highlighted that sub-millimeter movements of the electrodes caused by the robotic perturbation, while tolerable in normal laboratory conditions, produce artifacts up to 5–10 times larger than the stretch-evoked EMG response when using off-the-shelf electrodes in the static field of the MRI scanner (Fig. S6 in the Supplementary Materials).

These artifacts are a consequence of the Maxwell-Faraday law that describes how a time-varying magnetic flux through the surface enclosed by a conductive loop induces current on the conductive loop. For our specific application, the time-varying flux is generated because the path of the electrode leads during wrist perturbation unpredictably moves and deforms, leading to a change in the area enclosed by the conductive loop. Moreover, because the MRI static magnetic field is non-homogeneous in space, the amplitude of the artifact is highly dependent on the position that the electrodes occupy in space, which cannot be guaranteed to be constant during an experiment.

However, since motion-induced artifacts are ultimately a function of the position of electrodes and wire path over time, they could theoretically be removed by (1) co-locating reference electrodes (measuring only motion-induced artifacts) with measurement electrodes (measuring both motion-induced artifacts and muscle EMG), (2) matching the electrode-to-electrode and electrode-to-ground impedance of the two sets of electrodes, (3) routing the leads of the reference and measurement electrodes so that their path is the same.

We have developed a set of electrodes that embodies these principles to measure EMG signal of forearm muscles during fMRI (Fig. 1C). The set includes a bipolar reference electrode (REF) separated by a measurement electrode (EMG) via an insulating layer. Both pairs of electrodes are off-the-shelf Ag/AgCl bipolar electrodes (multitrode, Brain Products, Munich, Germany), with the terminals of the EMG electrode that are placed on the belly of the corresponding muscle and aligned with the direction of the muscle fiber. The REF electrode pair, colocated with the EMG pair, is then embedded in a conductive substrate (Squishy Circuits, Anoka, MN, USA) that approximates the electrode-to-electrode and electrode-to-ground impedance of superficial forearm muscles (2–5 M$\Omega$). To obtain the best matching wire path between REF and EMG electrode leads, all wires are routed through a StarQuad cable (VDC 268-026-000, Van Damme Cable). Proper coiling around a central core in a StarQuad cable ensures that differential signals of the two sets of bipolar electrodes carry the same motion-induced signal of a virtual conductor routed through the center of the core. The conductive substrate was then grounded using the shield terminal of the StarQuad cable to the amplifier ground, and connected to a fifth Ag/AgCl electrode placed on the lateral epicondyle of the elbow. A similar approach has been used to minimize fMRI-induced artifacts on EEG recordings^30^.

Because perfect (i.e. whole spectrum) impedance matching cannot be guaranteed, a simple subtraction of the EMG and REF signals would not ensure sufficient compensation of motion artifacts. As such, we developed a novel processing scheme that uses adaptive noise cancellation (ANC) to estimate artifact-free EMG signal (Fig. 1D). ANC is an adaptive filtering technique based on a multi-layer neural network that can be used to estimate a signal *x* corrupted by an interference *w*^31^. To properly work, the ANC takes two inputs consisting of the corrupted signal $y = x + w$ and a measurement of one or more interference signals ${\mathbf {r}}$. Considering that the true interference is altered when passing through two different measurement channels, signals ${\mathbf {r}}$ and *w* may be non-linearly related, such that a simple subtraction between *y* and components of ${\mathbf {r}}$ usually causes the distortion of the estimated signal ${\hat{x}}$. To solve this problem, ANC attempts to learn the non-linear relationship $f({\mathbf {r}})$ between the interference measured by the two different channels, estimating the signal ${{\hat{w}}} = f({\mathbf {r}})$, so that the desired signal can be estimated as ${{\hat{x}}} = y - {{\hat{w}}} = x +w - {{\hat{w}}}$.

For our analysis, we implemented the ANC scheme in MATLAB 2019a (MathWorks Inc., Natick, MA, USA) using an Artificial Neural Network Fuzzy Inference System (ANFIS)^32^. After appropriate learning, ANFIS constructs the non-linear mapping for the input-output relationship (estimate *x* given ${\mathbf {r}}$ and *y* in our case) without requiring a-priori knowledge of the structure of $f({\mathbf {r}})$. Learning occurs by tuning a set of membership functions that refer to the different layers of the network. In our algorithm, we used a hybrid iterative optimization method consisting of back-propagation for the parameters associated with the input membership functions, and least squares estimation for the parameters associated with the output membership functions^32^. Since the model structure has a large number of parameters, there is a risk for the ANFIS to overfit the data. To avoid overfitting, the algorithm partitions at every iteration a random sample from the entire dataset (in our case, a single continuous random interval within the time-series of *y* and ${\mathbf {r}}$ collected during a given perturbation) and uses it to cross-validate the model. The idea is that the cross validation error decreases until overfitting starts to occur. As such, the algorithm selects the set of parameters for the membership functions that refer to the solution with the minimum cross-validation error^32^.

In our implementation, we considered the interference signals ${\mathbf {r}}$ as a three-component vector, including the interference signal *r* measured by REF electrodes, its derivative $\dot{r}$, and a signal that quantifies the perturbation-related motion, as measured by the rotative encoder built in the MR-SW ($\theta$). The ANFIS was implemented using the *genfis* and *anfis* functions built in the Fuzzy Logic Toolbox.

### Procedures

#### Population

27 healthy individuals (16 males, 11 females; age range 19–38 years) volunteered to participate in one of the two experiments. 13 participants were involved in Experiment 1, 14 participants were involved in Experiment 2. All participants self reported as right handed, free from neurological disorders, orthopedic or pain conditions affecting the right arm and provided informed written consent prior to data collection. This study was approved by the Investigation Review Board of the University of Delaware Protocol no. 1097082-5 and was conducted in accordance with the Declaration of Helsinki.

Of the 27 participants recruited for this study, 25 (12 for Experiment 1 and 13 for Experiment 2) completed the full experimental protocol. Two participants only completed one of the two fMRI sessions because of discomfort during imaging. For this reason, they were excluded from the all imaging analyses. Due to technical issues affecting the quality of data collected during the experiments, other exclusions were required. Because of faulty EMG recordings (disconnected electrode wire) during fMRI, data collected from three participants (all involved in Experiment 2) were excluded from all the analyses described above. Moreover, because of errors in slice prescription, imaging data collected in three other participants (all involved in Experiment 1) were excluded from the fMRI analyses.

As a result, the EMG validation analysis was performed on data collected from $n=12$ individuals (all involved in Experiment 1), while the fMRI analyses were performed on $n=18$ participants (9 participants involved in Experiment 1, 9 participants involved in Experiment 2).

#### Experiment 1

Experiment 1 was composed of a total of four sessions, all performed during the same visit. The first and last sessions ($\text {OUT}_1$ and $\text {OUT}_2$) were performed in a mock scanner outside the MRI room, allowing the participant to maintain a supine posture similar to the one required for fMRI scanning. This was done in the attempt to replicate imaging conditions as much as possible, while removing any source of interference induced by the MRI electromagnetic fields. The two middle sessions ($\text {IN}_1$ and $\text {IN}_2$) were performed inside the MRI scanner (Siemens Prisma 3T scanner using a 64 channel coil), during fMRI. Parameters used for the MRI sequence included: Multi-Band Accelerated EPI Pulse sequence; 2 × 2 × 2 mm^3^ voxel resolution with 0.3 mm slice spacing; 46 deg degree flip angle; 110 × 110 px per image, 60 slices; TR = 1225 ms; TE = 30 ms; pixel bandwidth = 1625 Hz/pixel; receiver gain: high; simultaneous multi slice acceleration factor: 4. After the two functional sessions, a high resolution structural scan (magnetization-prepared rapid acquisition with gradient echo (MPRAGE): 0.7 × 0.7 × 0.7 mm^3^ resolution, with TR = 2300 ms, and TE = 3.2 ms; 160 slices with 256 × 256 px per image, was acquired for registration and normalization of the results to a common space. The full experimental protocol took about 140 min divided as 45 min for the robot setup and the electrode placement, 20 min per each one of the four experimental sessions, and 15 min for the structural scan.

#### Experiment 2

Experiment 2 was composed by only sessions $\text {IN}_1$ and $\text {IN}_2$ performed in Experiment 1—i.e. those performed during fMRI scanning. The full experimental protocol took about 85 min divided as 30 min for the robot setup and the electrode placement, 20 min per each one of the two experimental sessions, and 15 min for the structural scan.

#### Protocol

In all conditions, participants were exposed to a sequence of Ramp-and-Hold (RaH) perturbations in either flexion or extension at multiple velocities while EMG was recorded from the flexor carpi radialis (FCR) and the extensor carpi ulnaris (ECU). Before the perturbation onset, participants were visually cued to apply 200 mNm of background torque in the direction opposing the ensuing perturbation (e.g. if the perturbation was in the extension direction the participant was required to apply a flexion torque) to condition the muscles that would be stretched by the perturbation. The perturbation was then automatically triggered after the error between the target and measured torque was below 25 mNm for a given time $\text {T}_{\text {hold}}$. To avoid habituation to the time delay, for each perturbation the time $\text {T}_{\text {hold}}$ was randomly selected between 400 ms and 800 ms. Because the impact dynamics that characterize the end of a perturbation generates undesired oscillations in the EMG recording, leading to detection of false positive activity in the LLR window, perturbations were kept active for a fixed time of 200 ms, regardless of the magnitude of perturbation velocity. A rest period lasting for an interval randomly selected between 7 and 10 s was then included after each perturbation, after which the robot slowly moved the hand back to the neutral position with a return velocity set to 25 deg/s. Additional 4 s of rest were then allowed before the toque target for the following perturbation was displayed to the participant. Participants were instructed to yield to all perturbations.

In each session, participants were exposed to a total of 60 perturbations, pseudo-randomly selected from a pool of six different perturbation velocities ([50 125 200] deg/s in flexion or extension) repeated 10 times each. Before the beginning of each session, participants were visually cued to apply and hold a set of ten interleaved flexion and extension isometric torques. In both directions, the magnitude of the desired torque was set to be 500 mNm, and the participants were asked to keep it constant with a maximum error of 25 mNm for 5 s. This set of contractions was used to normalize stretch-evoked responses and enable group analysis of collected EMG data.

### EMG acquisition and analysis

Surface EMG was recorded with the BrainVision Recorder software (Brain Products, Munich, Germany) using a 16-channel MR-compatible bipolar amplifier (ExG, Brain Products, Munich, Germany). Given our interest in studying the neural substrates of LLR for both flexors and extensors, EMG was recorded from two wrist muscles: Flexor Carpi Radialis (FCR) and Extensor Carpi Ulnaris (ECU).

For each muscle, after having carefully cleaned the skin with a 70% Isopropyl Alcohol solution, we placed the bottom layer of electrodes on the belly of the muscle oriented along the muscle fiber, and filled the central hole of each electrode with an abrasive Electrolyte-Gel (Abralyt HiCl, Rouge Resolution, Cardiff, UK). Contact impedance for each electrode was measured using the BrainVision Recoder software and a cotton swab dipped in abrasive gel was swirled on the skin until the measured contact impedance was lower then $10\,\text {k}{\Omega }$, as described in the product technical specification. We then carefully co-located the reference electrodes on top of the measurement electrodes, using a layer of electric tape applied on top of the measurement electrodes to avoid electrical contact. Finally, we placed the conductive substrate on top of the reference electrodes and connected it to ground. In order to minimize relative motion of the different components of the apparatus, we applied pre-wrap around the entire forearm.

EMG data have been processed using three different pipelines to compare the novel processing scheme presented in this paper with two standard methods. The first method (STD) relies on the assumption that MRI-related movement artifacts are negligible and so it implements the same standard pipeline used to process the data recorded outside the scanner (Fig. S7A). In this way, the estimate of the EMG signal ${{\hat{x}}}$ is considered to be ${{\hat{x}}} = y$. The second method (SUB) compensates for MR-related movement artifacts assuming perfect match between the interference *r* measured by the REF electrodes and the true interference *w* (Fig. S7B). As such, it quantifies the EMG signal as ${{\hat{x}}} = y - r$. Finally, the third method fully implements the pipeline described in “StretchfMRI technique” section, quantifying the EMG signal as ${{\hat{x}}} = y - {\hat{f}}({\mathbf {r}})$ (Fig. 1D).

The EMG signal was processed to quantify reflex responses using standard pipeline^33^ modified to include the ANC and SUB pipelines. Specifically, both REF and EMG signals were initially segmented to extract the subset of data points representing perturbation-related activity recorded during the 200 ms silent window (25 ms after volume acquisition is completed), so that the first time point would correspond to the perturbation onset. The segmented signals were band-pass filtered using a 4th order Butterworth filter with cut-off frequencies $f_{LP} =20 \text { Hz}$, and $f_{HP} =250 \text { Hz}$, and fed to the later components of the filtering pipeline (Fig. 1D, for ANC this is signal $y_f$). The estimate of the EMG activity returned by the ANC, SUB, and STD filters was finally rectified and low-pass filtered with a 4$^{\text {th}}$ order Butterworth filter with cut-off frequency $f_{ENV} =60 \text { Hz}$. To allow between-subject comparison, after filtering, we normalized the stretch-evoked EMG activity by the average EMG ($\overline{EMG_c}|_j$) measured during the isometric contractions of the muscle *j* recorded prior to the beginning of each perturbation session. To determine $\overline{EMG_c}|_j$, we used only the central 3 s of activity recorded for the subset of contractions in which the given muscle was active—i.e. only the flexion torques for the FCR and only the extension torques for the ECU. The same constant was used to normalize EMG activity measured in response to perturbations that both stretched and shortened the muscle. Finally, to extract the magnitude of the long-latency response $H_{i,j}$ elicited by perturbation *i* on muscle *j*, we used the *cumsum* method^34^, quantifying $H_{i,j}$ as the area underlying the processed EMG signal $EMG_{i,j}(t)$ in the time window [50, 100] ms after the perturbation onset:

1 $$\begin{aligned} H_{i,j} = \int _{50}^{100} EMG_{i,j} dt \end{aligned}$$

where time is expressed in ms.

### Validation of the EMG measurements during fMRI

To validate the ability of StretchfMRI to reliably condition and quantify stretch-evoked LLR responses during fMRI, we used the EMG data recorded in the four sessions of Experiment 1. As no MR-related noise is expected in the sessions performed outside the MRI scanner, we considered the sessions $\text {OUT}_1$ and $\text {OUT}_2$ to act as a gold standard of measured stretch-evoked responses that is reflective of the true LLR responses, useful for comparison with the LLR-related activity measured during sessions $\text {IN}_1$ and $\text {IN}_2$.

EMG signals recorded during the two OUT sessions were processed using a standard pipeline that used all steps described above, but only used the STD filter (no expected signal from REF electrodes); while the EMG recorded during the sessions performed inside the MRI scanner was processed using the three different pipelines described in “EMG acquisition and analysis”.

Finally, for all sessions ($s = \left[ \text {OUT}_1, \text {IN}_1, \text {IN}_2, \text {OUT}_2 \right]$), filtering pipelines ($f = \left[ \text {STD}, \text {SUB}, \text {ANC} \right]$), and participants ($p = \left[ \text {P}01, \text {P}02, ..., \text {P}27 \right]$), we computed the amplitude of the stretch-evoked muscle activity $H_{i,j,v}|_{s,f,p}$ for all repetitions ($i = \left[ 1,2,...,10\right]$), both muscles $j = \left[ \text {FCR}, \text {ECU}\right]$, and all perturbations velocities ($v = \left[ -200, -125, -50, 50, 125, 200\right]$ deg/s) using Eq. (1). In general, averages across repetitions are indicated as ${\bar{H}}_{j^{*},v^{*}}|_{s^{*},f^{*},p^{*}}$ for specific levels of all other factors. The bold notation ${\bar{\mathbf{H}}}$ will be used to refer to the set of ${\bar{H}}$ measured for all levels of one or more factors. When this operation is performed, the indices of the corresponding factors are removed (e.g. ${\bar{\mathbf{H}}}|_{IN_1, ANC}$ refers to the set of the mean LLR amplitudes measured during the session $\text {IN}_1$ in both muscles for all participants and all perturbation velocities, when the ANC filter is used).

#### Statistical analysis

We quantified the accuracy afforded by each filtering method in identifying the true stretch-evoked muscle responses during fMRI at both the group level (combining measurements at all levels of factors muscle, velocity, participant), and at the individual perturbation level (considering each level of factors muscle, velocity, and repetition separately). With the group level analysis, we sought to quantify the agreement between the average LLR amplitude (${\bar{\mathbf{H}}}$) measured inside and outside the MRI for each filtering method. With the perturbation-specific analysis, we sought to quantify the deviation between each stretch-evoked response measured during MRI and the distribution of responses measured in the $\text {OUT}_1$ session in the same subject, velocity, and muscle. The analysis presented below is specific to the fMRI session $\text {IN}_1$; the results obtained when the following methods are applied to the session $\text {IN}_2$ are reported in the Supplementary Materials.

##### Group level analysis

For the group level analysis, we used the paired Bland-Altman (BA) analysis^35^, a statistical method to assess agreement between two measurement techniques when they are used to obtain two sets of data points in paired experimental conditions. Specifically, we compared the group level sets ${\bar{\mathbf{H}}}|_{IN_1,f}$ and ${\bar{\mathbf{H}}}|_{OUT_1}$ pairing the average LLR response measured for each perturbation velocity, each muscle, and for each participant. Due to the difference between responses obtained as a consequence of muscle stretch and shortening, we considering separately the subset of values measured in response to muscle stretch (${\tilde{\mathbf{H}}}|_f^{st}$) and shortening (${\tilde{\mathbf{H}}}|_f^{sh}$).

For each of the two stimulus direction conditions (*d*) (i.e. stretch and shortening), we used BA analysis to determine bias (B$|^{d}_{\text {IN},f}$) as the mean difference between paired measurements ${\tilde{\mathbf{H}}}|^d_{IN_1,f} - {\tilde{\mathbf{H}}}|^d_{OUT_1}$, for all conditions, and the 95% limits of agreement (LoA$|^{d}_{\text {IN},f}$), defined as the interval where given one measurement, we expect to measure the other in 95% of the cases. Each metric is measured with its own precision, expressed in terms of its 95% confidence interval. Both bias and limits of agreement are expected to decrease with an increase in agreement between the two techniques. However, when the paired measurements are not obtained simultaneously with two techniques, the metrics of test-retest reliability extracted by BA analysis will be affected by both measurement error and by intrinsic physiological variability of the measurand, in this case stretch-evoked responses^20^. As such, bias or limits of agreement may be artificially inflated by physiological variability. To isolate the effects of measurement error from those of physiological variability, we thus defined our outcome measures as contrasts between the reliability measured via the $\text {IN}_1$ vs. $\text {OUT}_1$ comparison and the reliability measured via the $\text {OUT}_2$ vs. $\text {OUT}_1$ comparison.

We thus used the metrics obtained with the BA analysis to make inference on *(i)* whether filtering method affects test-retest reliability, and *(ii)* whether filtering method affords test-retest reliability comparable with the baseline variability of the physiological process being measured (obtained for the $\text {OUT}_2$ vs. $\text {OUT}_1$ comparison).

We tested the null hypothesis that the estimated bias does not change when using different filtering methods by calculating the 95% confidence interval of bias for the $\text {IN}_1$ vs. $\text {OUT}_1$ comparison for each filtering method (B$|^{d}_{\text {IN},f}$), and performing three pairwise comparisons of the resulting confidence intervals (one for each pair of methods), using the Bonferroni method to correct for multiple comparisons. Moreover, we tested the null hypothesis that the bias measured in the $\text {IN}_1$ vs. $\text {OUT}_1$ comparison was equal to the one measured in the REF comparison ($\text {OUT}_2$ vs. $\text {OUT}_1$) by establishing if any of the intervals (B$|^{d}_{\text {IN},f}$) overlapped with the confidence interval (B$|^{d}_{\text {REF}}$) measured for the $\text {OUT}_2$ vs. $\text {OUT}_1$ comparison.

We conducted a similar analysis for the LoA metrics to make inference on the test-retest reliability of a specific measurement. Because the LoA is defined as an interval, test-retest reliability inferences are based on the analysis of the Jaccard index, used to quantify the relative overlap of two intervals as the ratio between their intersection and union. In our case, the Jaccard index $J|_f^{d}$ is defined as a function of the LoA measured for the comparison $\text {IN}_1$ vs. $\text {OUT}_1$ (LoA$|^{d}_{\text {IN},f}$), and the LoA measured for the comparison $\text {OUT}_2$ vs. $\text {OUT}_1$ (LoA$|^{d}_{\text {REF}}$), as

2 $$\begin{aligned} \text {J}|^{d}_{f} = \frac{\text {LoA}|^{d}_{\text {IN},f} \cap \text {LoA}|^{d}_{\text {REF}}}{\text {LoA}|^{d}_{\text {IN},f} \cup \text {LoA}|^{d}_{\text {REF}}} \end{aligned}$$

With this procedure, the test-retest error afforded by each filtering method was scaled between 0 and 1, with a score of 1 representing a perfect match of the LoA in the two conditions. Because each LoA is measured with its own precision, we determined the 95% confidence intervals for the Jaccard index using bootstrapping to numerically compute the distribution of Jaccard indices when the LoA for the comparisons $\text {IN}_1$ vs. $\text {OUT}_1$ and $\text {OUT}_2$ vs. $\text {OUT}_1$ were randomly sampled from a normal distribution with mean and standard deviation calculated from the BA analysis coefficients.

Similarly to the estimated bias analysis, we used the estimated distributions of Jaccard coefficients to make inference on whether filtering method affects test-retest reliability. Specifically, we tested the null hypothesis that there is no difference in the overlap of the limits of agreement by establishing if the 95% confidence interval of Jaccard indices overlapped for different levels of filtering method (three comparisons).

##### Perturbation-specific analysis

The goal of the perturbation-specific analysis was to quantify the departure of each stretch-evoked response measured during fMRI from the baseline values measured during the $\text {OUT}_1$ session. To this aim, we computed the standardized z-score separately for each value in the set $\mathbf{H}|_{IN_1, f}$ as:

3 $$\begin{aligned} z_{i,j,v}|_{IN_1,f,p} = \frac{H_{i,j,v}|_{IN_1,f,p}-\mu _{j,v}|_{p} }{\sigma _{j,v}|_{p} } \end{aligned}$$

where *i*, *j*, *v*, *f*, and *p* are the perturbation, muscle, velocity, and participant indices, respectively; while ($\mu _{j,v}|_{p}$) and ($\sigma _{j,v}|_{p}$) are the population mean and standard deviation of the set $\mathbf{H}_{j,v}|_{OUT_1,p}$. To simplify the interpretation of the results, for each stimulus direction condition *d*, we determined the standard deviation ($\sigma _{v}|^{d}_{IN_1,f}$) of the values in the set $\mathbf{z}_{v}|^{d}_{IN_1,f}$. Ideally, if the elements in the sets $\mathbf{H}_{v}|^{d}_{IN_1, f}$ and $\mathbf{H}_{v}|^{d}_{OUT_1}$ are sampled from the same normal distribution, the standard deviation $\sigma _{v}|^{d}_{IN_1,f}$ should have unitary magnitude. However, due to the physiological variability of the stretch-evoked muscle response the distribution of the elements in the two sets might have different standard deviation. As such, to have a term of comparison, we determined also the standard deviation $\sigma _{v}|^{d}_{REF}$ of the set of standardized scores $\mathbf{z}_{v}|^{d}_{REF,f}$ obtained applying Eq. (3) to the elements in the set $\mathbf{H}_{j,v}|_{OUT_2,p}$.

We then tested the null hypothesis that the variance of the sets $\mathbf{z}_{v}|^{d}_{IN_1,f}$ is the same for all filtering methods, performing three Bartlett tests, one for each pair of sets $\mathbf{z}_{v}|^{d}_{IN_1,f_i}$, $i=1,2,3$. Moreover, we performed three additional Bartlett tests to test the null hypothesis that the variance of the sets $\mathbf{z}_{v}|^{d}_{REF}$ and $\mathbf{z}_{v}|^{d}_{IN_1,f}$ is the same for each filtering method (one test per each filtering method). The Bonferroni correction was used to control for multiple comparisons when multiple tests were applied to the same outcome measures.

### Statistical analysis of imaging data

We performed the analysis of all fMRI datasets using SPM12 (Wellcome Department of Cognitive Neurology, London, UK, http://www.fil.ion.ucl.ac.uk/spm) running on Matlab 2019a (Mathworks, Inc., Natick, MA, USA, http://www.mathworks.com). Functional datasets were preprocessed using a standard pipeline composed of five steps: realignment to the mean image, co-registration to structural MPRAGE, normalization to a standard MNI space template, spatial smoothing, and high-pass filtering (cut off at 128 s). For spatial smoothing, we used a Gaussian kernel with FWHM = 4 mm for voxels in the brainstem for brainstem-specific analyses (both hypothesis-testing and reliability analyses), and FWHM = 8 mm for the all other analyses. 4 mm smoothing of the brainstem was selected to account for the different expected extent of functional activations of brainstem nuclei compared to whole brain, and was used only for testing a regionally-specific (brainstem) hypotheses.

For each scanning session, we performed two first-level analyses to determine activation in the whole brain and in the brainstem, separately. While we used the same general linear model (GLM) for both analyses, we convolved it with two different hemodynamic response functions (HRF) to account for different hemodynamic response dynamics expected in different brain regions. A brainstem-specific HRF^36^ (delay of the peak = 4.5 s, delay of the undershoot = 10 s, dispersion of response = 1 s, dispersion of undershoot = 1 s, ratio of peak to undershoot = 15, length of kernel = 32 s) was used to assess activation in the brainstem, while a standard HRF (delay of the peak = 6 s, delay of the undershoot = 16 s, dispersion of response = 1 s, dispersion of undershoot = 1 s, ratio of peak to undershoot = 6, length of kernel = 32 s) was used to assess activation in all other regions. The neural response was modeled as:

4 $$\begin{aligned} \mathbf {y} = \beta _{\text {FCR}} \mathbf {H}_{\mathbf {FCR}}^{\star } + \beta _{\text {ECU}} \mathbf {H}_{\mathbf {ECU}}^{\star } + \beta _0 + \varvec{\beta }_{\mathbf {R}} \mathbf {R}, \end{aligned}$$

where $\mathbf {H}_{\mathbf {FCR}}^{\star }$ and $\mathbf {H}_{\mathbf {ECU}}^{\star }$ are obtained by convolution of rectangular functions (duration: 50 ms, onset: 50 ms after perturbation start, amplitude: ${\tilde{\mathbf {H}}_{FCR}|_{ANC}^{st}}$ and ${\tilde{\mathbf {H}}_{ECU}|_{ANC}^{st}}$, respectively) with the appropriate HRF (Fig. S8 in the Supplementary Material) to account for BOLD signal associated with LLRs of specific muscles. As such, the regressors of interest accounted for the magnitude of LLR amplitude measured for each muscle under stretch. An additional set of 6 nuisance regressors ${\mathbf {R}}$ was included to account for variance in the measured signal associated with 3D head movements (translation and rotation).

To determine voxels whose BOLD signal was significantly associated with LLRs of specific muscles under stretch, we first calculated the statistical maps for the contrasts $\beta _{\text {FCR}} > 0$ and $\beta _{\text {ECU}} > 0$ for each subject, and then used the contrast images as input for the second level analysis used to determine a group level effect.

Because participants were asked to contract their muscles prior to each perturbation, there is an intrinsic statistical association between the muscle state before the perturbation and the perturbation itself. As such, given the small temporal resolution of fMRI, it is possible that some of the variance in the BOLD signal explained by LLR-related regressors might actually arise from the neural activity associated with the background contraction. To rule out this concern, we conducted two alternative analyses: one based on simulated data, and one based on the implementation of a different GLM to account for neural signal associated with background muscle activity. A detailed description of these analyses and the respective discussion are included in the Supplementary Materials.

### Test-retest reliability of neural activations

Data collected in two consecutive fMRI sessions ($\text {IN}_1$ and $\text {IN}_2$) were used to establish the test-retest reliability of fMRI data in different brain regions. Reliability of the activation maps was quantified at both the individual subject and group levels. For both levels of analysis we quantified reliability for the whole brain and for one bilateral and eight unilateral anatomical Regions of Interest (ROIs) known to be associated with the execution of motor actions: primary motor cortex (M1), primary somatosensory cortex (S1), premotor cortex (PM), superior parietal lobule (SPL), intraparietal sulcus (IPS), cerebellum (Cr), thalamus (Th), putamen (Pt), and brainstem (Bs). For each unilateral ROI, we considered two separate ROIs that refer to the left and right hemispheres. The specific brain areas included in each ROI are reported in Table 1 and were obtained by thresholding the Juelich, Harvard Subcortical, or Cerebellar MNI152 brain atlases at a probability of 50%.

**Table 1:** Definition of ROIs for test-retest reliability analysis.

ROI		Brain areas	
Name	Acronym	Name	Atlas
**Cortical ROIs**			
Primary motor cortex	M1	BA4a, BA4b	Juelich atlas
Premotor cortex	PM	BA6	
Primary somatosensory cortex	S1	BA1, BA2, BA3a, BA3b	
Superior parietal lobule	SPL	BA5ci, BA5l, BA5m,	
		BA7a, BA7m, BA7p, BA7pc	
Intraparietal sulcus	IPS	hIP1, hIP2, hIP3	
**Cerebellar ROI**			
Cerebellum	Cr	Crus 1, Crus2	Cerebellar MNI152 atlas
		CR I-VI, VIII, IX	
**Subcortical ROIs**			
Thalamus	Th		Harvard subcortical atlas
Putamen	Pt		
Brainstem	Bs		

For each region listed above, with the exception of the Brainstem, we created two ROIs for the left and right hemisphere.

BA: Brodmann Area, CR: Cerebellar Region, hIP: human IntraParietal Area.

To quantify spatial congruence between the thresholded t-maps (p < 0.001, uncorrected) obtained for the two sessions $\text {IN}_1$ and $\text {IN}_2$, we used the Sørensen-Dice index^37^:

5 $$\begin{aligned} S = \dfrac{2 V_o}{V_1 + V_2} \end{aligned}$$

where $V_o$ represents the number of supra-threshold voxels contained in both $\text {IN}_1$ and $\text {IN}_2$, while $\text {V}_1$ and $\text {V}_2$ represent the number of supra-threshold voxels contained in $\text {IN}_1$ and $\text {IN}_2$, respectively. This index ranges between 0 and 1, with 0 meaning no overlap and 1 meaning perfect overlap. Activation maps resulting from the first- and second-level analyses have been used to calculate the Sørensen-Dice index at the individual subject ($\text {S}_{{p}}$) and at the group levels ($\text {S}_g$), respectively.

While the Sørensen-Dice index is commonly used to quantify test-retest reliability of pair of fMRI-based activation maps, the operation of thresholding used to calculate it loses a lot of information that could be relevant to quantify test-retest reliability. To overcome these limitations, we complemented the Sørensen-Dice index with a second index, the intraclass correlation coefficient (ICC)^38,39^. We performed two different analyses to determine the ICC both at the individual participant ($\text {ICC}_p$) and at the group levels ($\text {ICC}_g$). For the participant-specific analysis, we calculated the ICC applying the definition of the ICC(3,1) proposed by Shrout and Fleiss^40^ to the subset of voxels contained in a specific ROI (Table 1) that, in the case of only two repeated measurements, simplify to:

6 $$\begin{aligned} \text {ICC}(3,1) = \dfrac{BMS-EMS}{BMS + EMS} \end{aligned}$$

where BMS is the Between voxel Mean Square variance and EMS is the Error Mean Square variance obtained using a two-way mixed effect ANOVA, with voxel as a random effect, and session as a fixed effect.

For the group level analysis, we calculated reliability maps applying Eq. (6) to each voxel in the brain assuming subject as a random effect and session as a fixed effect. To obtain the ROI-specific $\text {ICC}_g$, we computed the median of the ICC distributions within each region.

For each of the two subject-specific reliability indices ($\text {S}_p$ and $\text {ICC}_p$), we performed statistical inference to test two separate null hypotheses: $h_0|^1$) within each ROI, the means of the reliability indices obtained for the two regressors ($\mathbf {H}_{\mathbf {FCR}}^{\star }$ and $\mathbf {H}_{\mathbf {ECU}}^{\star }$) are equal; $h_0|^2$) for each regressor, the means of the reliability indices obtained for the unilateral ROIs are equal in the two hemispheres. To test $h_0|^1$, for each of the two indices, we performed 18 paired t-tests (one per ROI plus one for the whole brain). To test $h_0|^2$, for each of the two indices, we performed 16 paired t-tests (two per each tested ROI).

### Neural correlates of LLRs

#### Brainstem-specific analysis

We conducted our primary fMRI analysis to test the hypothesis that BOLD signal in any voxel in the brainstem was significantly associated with LLRs for flexors and/or extensor muscles under stretch. For this analysis, we combined data measured in sessions $\text {IN}_1$ and $\text {IN}_2$ by concatenating time-series measured in the two sessions, and adding a separate regressor to model the factor session. We used FWHM = 4 mm and the brainstem-specific HRF for this analysis. Given the regionally-specific nature of our primary hypothesis, we only included signal from brainstem voxels and applied a small-volume correction^41^ that uses random field theory^42^ to control for the Family-Wise Error rate (FWE) for voxel-specific t-tests at the $\alpha <0.05$ level in the region-of-interest (number of voxels at the 2-mm isotropic resolution: 3314). To identify the location of clusters of activation in the brainstem, we used the Harvard Ascending Arousal Network Atlas^43^, the Brainstem Connectome Atlas^44^, in addition to non MNI-based anatomical brainstem atlases^45,46^.

#### Whole-brain analysis

We conducted a secondary analysis to test the hypothesis that BOLD signal in any voxel of the brain was significantly associated with LLRs for flexors and/or extensor muscles. For this analysis, we used FWHM = 8 mm for all voxels and the standard HRF. Given the non-specific nature of this secondary hypothesis (number of voxels tested at the group level: 228,365), we controlled for Family-wise Error rate (FWE) in the whole brain using random field theory^42^ to achieve $\alpha <0.05$ for all voxels in the brain and applied a cluster correction of $k=10$ (only clusters of activation larger than *k* voxels are accounted).

#### Participant-specific analysis

We first extracted participant-specific *t*-scores from the first level analysis quantifying activation in the center of the two most significant clusters at the group level (MNI coordinates: [8, − 42, 40] mm for the right medulla, and [− 8, − 30, 26] mm for the left pons), then selected individuals with the greatest difference in *t*-scores for FCR-specific activation (right medulla), and ECU-specific activation (left pons)—subj 7 and 18 respectively—and individuals with similar *t*-scores for the two regressors—subjects 9 (right medulla) and 8 (left pons) (Fig. 6). Then, we quantified the residual signal not explained by nuisance regressors (FCR residuals, nuisance regressors: ECU + head movements; ECU residuals, nuisance regressors: FCR + head movements) to evaluate muscle-specific responses of the BOLD signal. We finally segmented and averaged the residual BOLD signal corresponding to multiple perturbations stretching specific muscles. BOLD residuals are overlaid with the regressor of interest from model 1 (scaled by the estimated $\beta _i$ coefficient), in Fig. 6, and with scaled regressors from model 2 in Fig. S13. The background regressor in Fig. 6 has arbitrary amplitude and is only reported for visual comparison.

## Results

### Validation of StretchfMRI

#### Reliability of stretch-evoked EMG during fMRI

**Figure 2:**
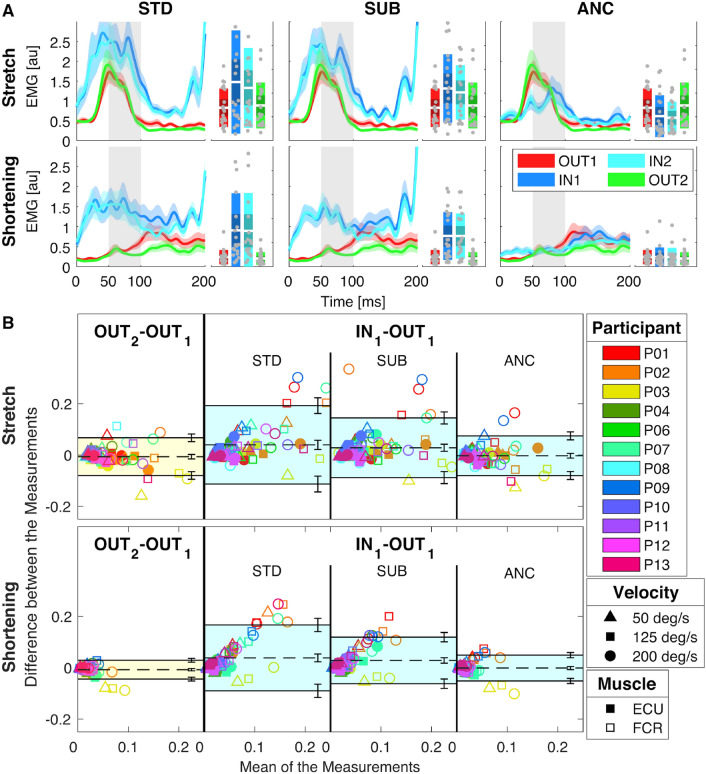
(**A**) Group-average of the stretch-evoked EMG signal measured using different processing pipelines in response to a 200 deg/s ramp-and-hold perturbation for both muscles undergoing stretch or shortening for Experiment 1. Plots are obtained using the same electrode set and different processing pipelines: standard (STD), a time-domain subtraction of measured and reference signals (SUB), and adaptive noise cancellation (ANC). In all graphs, the thick line represents the group mean and the shaded area the 95% confidence interval of the group mean. The gray shaded area indicates the time interval where an LLR is expected. Box plots indicate the distribution of LLR amplitude, with indications for the mean (white horizontal line), mean ± one st. dev. (dark shaded area), 95% confidence interval (light shaded area), with individual measurements overlaid. (**B**) Bland–Altman plots for the comparisons $\text {OUT}_2$ vs. $\text {OUT}_1$ (left) and $\text {IN}_1$ vs. $\text {OUT}_1$ (right) when different filtering pipelines are considered. Datapoints in each plot encode participants with colors, perturbation velocities with shapes, and muscles with filling style. The shaded area represents the interval between the negative and positive limits of agreement, while the dashed line indicates bias. Error bars on the side of each plot represent the 95% confidence intervals by which each parameter is estimated.

We validated the stretch-evoked EMG collected during fMRI by quantifying agreement between measurements collected inside and outside the MR scanner. We used two analyses: one based on the mean LLR responses averaged across multiple repetitions for each set of conditions (group-level analysis), and one based on the analysis of EMG measurements of individual perturbations (perturbation-specific analysis). Behavioral data from IN and OUT sessions are reported in the supplementary materials. For reference, the mean timeseries of the EMG recorded during Exp 1 and processed using three different filtering pipelines is shown in Fig. 2A. Muscle-specific EMG recordings are reported in Figs. S9, S10, respectively for FCR and ECU measured during Exp 1.

##### Group level results

Group-level analysis was performed using the Bland-Altman (BA) method^35^, with plots shown in Fig. 2B for the comparisons $\text {OUT}_2$ vs. $\text {OUT}_1$ and $\text {IN}_1$ vs. $\text {OUT}_1$. The values of bias, positive and negative limits of agreement (LoA), with the respective confidence intervals are reported in Table S8 in the supplementary materials.

**Figure 3:**
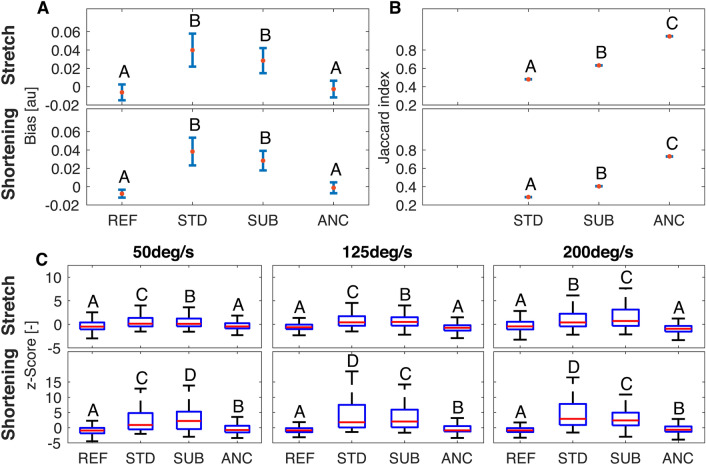
Mean and 95% confidence intervals for the bias (**A**), and the Jaccard index (**B**) obtained for the different filtering pipelines. Test-retest parameters measured for the two stimulus directions are reported in different rows. Letters indicate rankings of the test-retest parameters. Bars that do not share a letter have a statistically different mean. (**C**) Box plots representing the distributions of standardized *z*-scores measured for different combinations of perturbation velocity and filtering pipeline. Distributions obtained for the two stimulus directions are reported in different rows. Letters indicate rankings of the variance of each distribution; boxes that do not share a letter have a statistically different variance.

Based on the BA analysis, Adaptive Noise Cancellation (ANC) enables reliable estimation of LLR amplitude during fMRI sessions. In fact, the agreement between LLR amplitudes measured inside and outside the MRI is not worse than the agreement between two OUT sessions. Specifically, the bias estimated for the $\text {IN}_1$ vs. $\text {OUT}_1$ comparison was not significantly different from the one estimated for the $\text {OUT}_2$ vs. $\text {OUT}_1$ comparison (*z*(72)=0.56, $p = 0.57$ for stretch, $z(72)=1.70$ , $p=0.09$ for shortening). Additionally, the 95% confidence intervals of the bias estimated for both comparisons, in both muscle stimulus directions, intersect the zero value indicating that the measurement is unbiased (Fig. 3A). Finally, the overlap between the range defined by the LoA for two repeated OUT experiments and the range of LoA for IN vs. OUT experiment, quantified by the Jaccard coefficient, approached perfect overlap (mean ± s.e.m. $J=0.843 \pm 0.001$ for stretch, $J=0.783 \pm 0.001$ for shortening).

For reference, test-retest reliability was worse using standard filtering pipelines (STD and SUB). Specifically, the pairwise comparisons performed between the difference in measurements obtained when using different filtering pipelines rejected the null hypothesis for the comparisons STD vs. ANC and SUB vs. ANC, for both stretch (*z*(72) = 3.70, p < 0.001; *z*(72) = 4.13, p < 0.001, respectively) and shortening (*z*(72) = 4.75, p < 0.001; *z*(72) = 4.77, p < 0.001, respectively) (Fig. 3A). The measurements estimated using ANC were consistently smaller than the ones estimated using either STD or SUB (Table S8) in both shortening and stretch conditions. The ANC filter also afforded the highest overlap in LoA in quantifying LLRs during fMRI during both muscle stretch and shortening compared to other methods. Jaccard index values for ANC were greater than those obtained with the SUB filter ($J^{st}_{SUB}=0.593 \pm 0.001$, $J^{sh}_{SUB}=0.459 \pm 0.001$), and the STD filter ($J^{st}_{STD}=0.489 \pm 0.001$, $J^{sh}_{STD}=0.324 \pm 0.001$). Pairwise comparisons of the Jaccard indices rejected the null hypothesis for all comparisons showing a statistically significant difference between the test-retest error obtained using the different filtering pipelines, in both muscle stimulus conditions (p < 0.001 for all comparisons) (Table S8, Fig. 3B). The group level analysis has been repeated using measurements collected for the $\text {IN}_2$ session (Figs. S11, S12, and Table S10, supplementary materials), showing a perfect overlap of all statistically significant results.

##### Perturbation-specific results

With the perturbation-specific analysis, we sought to quantify the deviation between each stretch-evoked response during IN sessions and the distribution of responses measured in a matched reference (REF) condition ($\text {OUT}_1$ session), in terms of the *z*-score of each perturbation (Fig. 3C). A Bartlett test established that the variance in measurements collected during IN sessions using ANC was not significantly different from the one during OUT sessions in matched conditions for muscles undergoing stretch (50 deg/s: $\chi ^2 = 3.67$, *p* = 0.16; 125 deg/s: $\chi ^2 = 3.69$, *p* = 0.16; 200 deg/s: $\chi ^2 = 5.8$, *p* = 0.06), while the variance was greater during the IN condition for muscles undergoing shortening (50 deg/s: $\chi ^2 = 17.50$, p < 0.001; 125 deg/s: $\chi ^2 = 17.87$, p < 0.001; 200 deg/s: $\chi ^2 = 33.07$, p < 0.001).

For reference, the variance of *z*-scores measured using other filtering pipelines (STD and SUB) was greater than the one measured for both ANC and REF conditions ($p<0.001$ for all paired comparisons). Detailed figures and tables arising from this analysis are included in Table S9 of the supplementary materials. The perturbation-specific analysis is repeated using measurements collected for the $\text {IN}_2$ session (Figs. S11, S12, and Tabs. S10, S11 of the supplementary materials), showing a perfect overlap of all statistically significant results.

#### Test-retest reliability of neural activations

**Figure 4:**
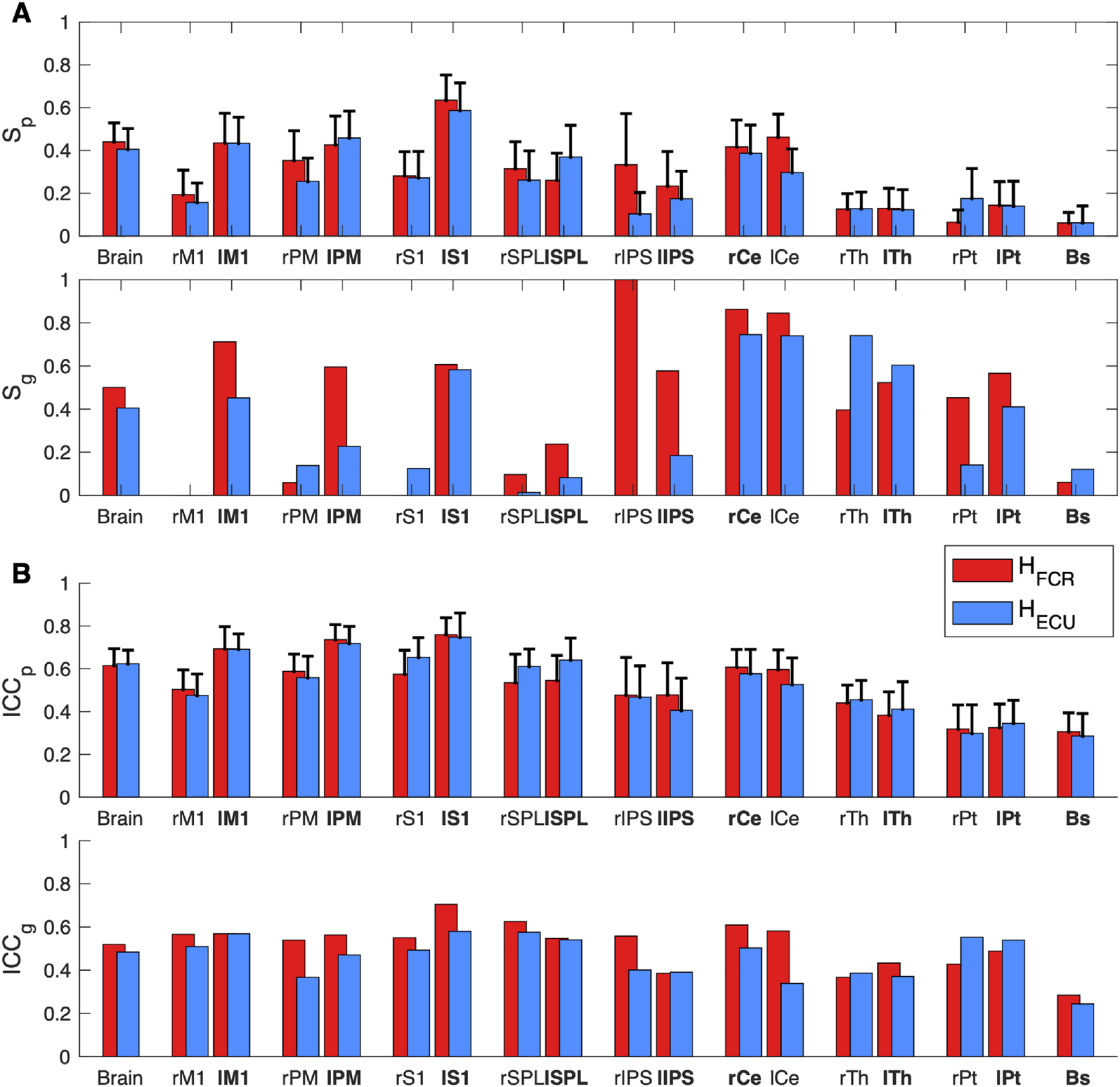
Test-retest reliability scores obtained in the whole brain and in the selected ROIs for the group level (g) and participant-specific (p) analyses. (**A**) Sørensen-Dice index, (**B**) Intraclass correlation coefficient. For $\text {ICC}_p$ and $\text {S}_p$, bar height indicates the mean value of the respective metric measured in all participants, with the error bars showing the 95% confidence intervals. Brain regions marked in bold are those regions were activation is expected.

The analysis of the overlap between the group level activation maps showed a moderate to good overlap in the whole brain as well as for all contralateral cortical ROIs for both regressors, with a poor or absent overlap in the ipsilateral ROIs (Fig. 4A and Table S12). Good to excellent overlap was observed bilaterally in the cerebellar ROI, while moderate overlap could be observed bilaterally in the Thalamus, in the left Putamen, and in the right Putamen for FCR-specific maps. Poor overlap could instead be observed in the right Putamen for the ECU-specific map and in the brainstem (Fig. 4A and Table S12). The analysis of participant-specific activation maps showed a moderate overlap in the whole brain as well as for all selected cortical and cerebellar ROIs, while fair to poor overlap can observed for the subcortical ROIs (Fig. 4A and Table S12). Similar reliability was measured for activation associated with flexor and extensor LLRs (paired *t*-test between subject specific Soerensen-Dice index $S_p$ failed to reject the null hypothesis $h_0|^1$ that they are the same for all selected selected ROIs with the exception of the right Putamen).

Additionally, the paired t-tests between the $\text {S}_p$ measured in the two hemispheres rejected the null hypothesis $h_0|^2$ for M1 (FCR: *t*(34) = − 2.71, *p* = 0.011; ECU: *t*(34) = − 3.44, *p* = 0.003), PM (FCR: *t*(34) = − 2.73, *p* = 0.010; ECU: *t*(34) = − 2.45, *p* = 0.020), and S1 (FCR: *t*(34) = − 2.66, *p* = 0.010), showing a significantly higher overlap in the contralateral sensorimotor cortex for both regressors. No significant difference was observed in any of the cerebellar and subcortical ROIs.

The ICC showed fair to good agreement between the session-specific statistical maps for the cortical and cerebellar ROIs both at the individual subject and the group level (Fig. 4B and Table S12). On the contrary, only poor to fair ICC can be observed in the subcortical ROIs (Fig. 4B and Table S12). The paired t-tests failed to reject the null hypothesis $h_0|^1$ for all ROIs, showing no significantly different test-retest reliability for the two regressors. Similarly to what was observed in the analysis of the overlap of the thresholded maps, the paired t-test rejected the null hypothesis $h_0|^2$ for M1 (FCR: *t*(34) = − 2.50, *p* = 0.018; ECU: *t*(34) = − 3.35, *p* = 0.002), PM (ECU *t*(34) = − 2.39, *p* = 0.022), and S1 (FCR: *t*(30) = − 3.99, *p* < 0.001; ECU: *t*(34) = − 3.41, *p* = 0.002), showing a significantly higher ICC in the contralateral sensorimotor cortex for both regressors. No significant difference was observed in any of the cerebellar and subcortical ROIs.

### Neural correlates of LLRs

#### Brainstem-specific analysis

**Figure 5:**
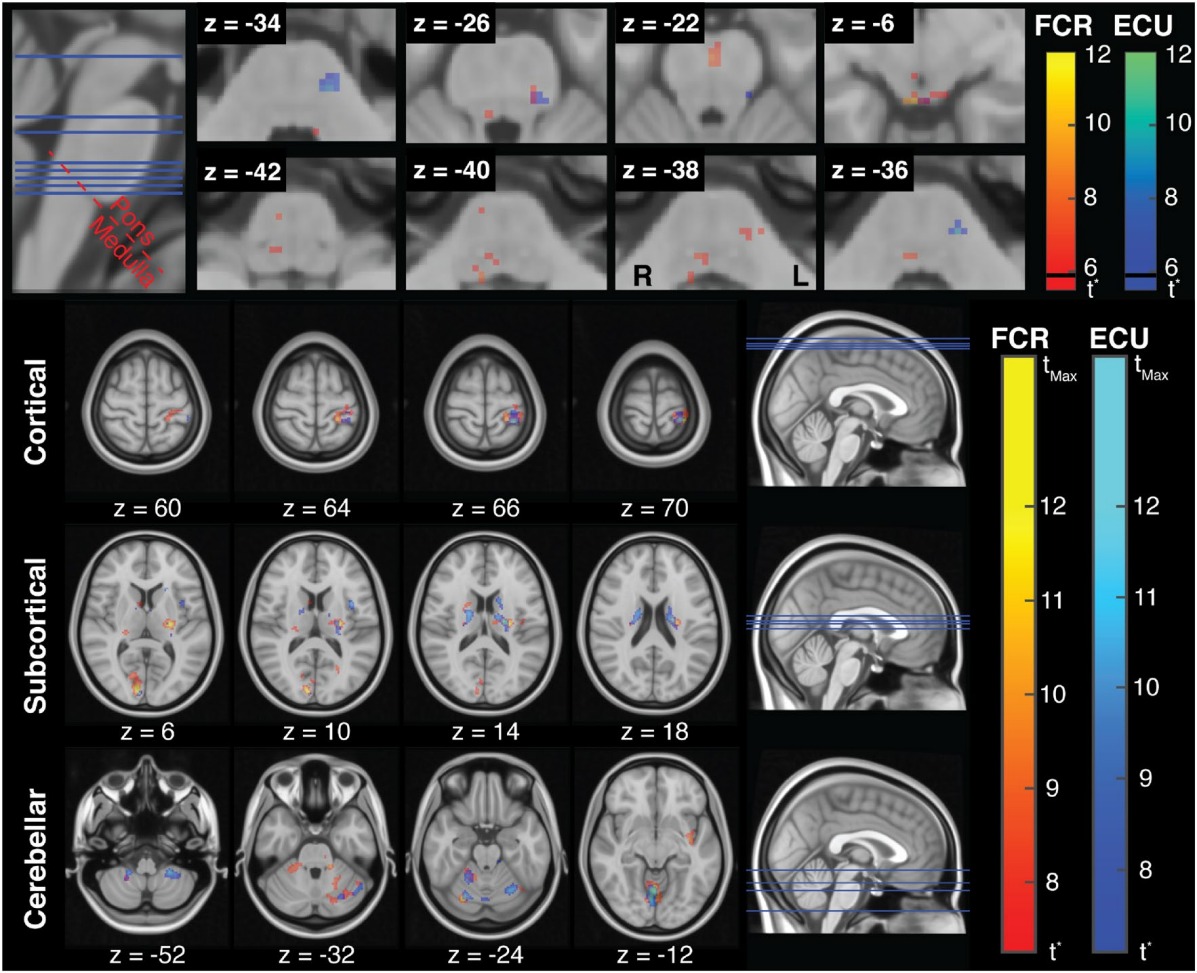
(**Top**) LLR-specific activation maps in the brainstem for FCR and ECU, with overlaid the plane of demarcation used to refer to voxels as being in the pons or in the medulla. The maps refer to contrasts $\beta _{\text {FCR}}>0$ and $\beta _{\text {ECU}}>0$ obtained for the brainstem-specific analysis. The threshold *t*-statistic, obtained after FWE correction, was equal 5.62 for both regressors. For reference, the *t*-statistic that refer to a Bonferroni correction ($\text {t}_{\text {Bon}}$= 5.8) is marked on each colorbar with a black line. (**Bottom**) LLR-specific activation maps in the whole-brain for FCR and ECU. The threshold *t*-statistic, obtained after FWE correction, was set to 7.25 and 7.09, respectively for FCR and ECU, while $\text {t}_{{Max}}$ was equal to 14.95 and 15.54, respectively. Colorbars are saturated at $t = 12$ for better visualization of *t*-statistic gradients. All statistical parametric maps are overlaid on axial slices of the standard Montreal Neurological Institute 152 template, with reported z coordinate in mm.

Muscle-specific activation maps included several clusters of activation in the brainstem. Specifically, LLR-related activity specific to FCR included a total of 57 voxels with supra-threshold *t*-scores, including three distinct clusters spanning bilaterally the midbrain, seven clusters spanning bilaterally the pons, and two clusters in the right superior medulla and inferior pons (Fig. 5 top and Table 2). LLR-related activity specific to ECU included a total of 25 voxels, including three clusters spanning bilaterally the midbrain, two clusters in the left pons and only one voxel in the right pons (Fig. 5 top and Table 2).

**Table 2:** Activation in the brainstem.

Brainstem region		Muscle	Peak-level		Cluster-level		Peak coordinate [mm]		
Area	Laterality		$\text {t}_{{max}}$	$\text {p}_{\text {FWE}}$	Voxels in cluster	$p_{\text {FWE}}$	x	y	z
Midbrain	R	FCR	10.22	< 0.001	21$^{\textit{a}}$	< 0.001	4	− 36	− 6
	R		5.93	0.026	2	0.009	4	− 30	− 4
	L		6.84	0.005	-Part of cluster *a*-		− 6	− 36	− 8
	L		5.62	0.049	1	0.019	− 6	− 30	− 2
	R	ECU	6.86	0.004	2	0.008	6	− 28	− 12
	R		6.21	0.015	2	0.008	2	− 36	− 6
	L		6.56	0.008	2	0.008	− 4	− 30	− 2
Pons	R	FCR	8.02	< 0.001	7	< 0.001	2	− 20	− 22
	R		6.62	0.007	1	0.019	6	− 26	− 42
	R		5.94	0.026	1	0.019	6	− 36	− 26
	R		5.81	0.033	1	0.019	8	− 22	− 40
	L		6.79	0.005	6$^{b}$	< 0.001	− 8	− 30	− 26
	L		5.95	0.025	-Part of cluster *b*-		− 8	− 24	− 24
	L		6.10	0.019	1	0.019	− 14	− 28	− 38
	L		6.00	0.023	3	0.004	− 8	− 28	− 38
	R	ECU	5.90	0.028	1	0.019	16	− 30	− 30
	L		9.12	< 0.001	12	< 0.001	− 10	− 28	− 36
	L		6.46	0.009	6	< 0.001	− 10	− 32	− 24
Medulla	R	FCR	7.45	0.002	5	< 0.001	8	− 42	− 40
	R		6.65	0.006	8$^{c}$	< 0.001	6	− 36	− 38
	R		5.75	0.037	-Part of cluster *c*-		8	− 36	− 42

Superscripts on the cluster volume, when present, refer to clusters with multiple peaks. Coordinates are express in MNI space.

There was nearly no overlap between the clusters of LLR-related activation and the Harvard Ascending Arousal Network Atlas^43^, an observation that suggests that the activation is indeed primarily in descending pathways in the brainstem. Also the amount of overlap between the main clusters of activation and the pathways included in the Brain Connectome Atlas^44^ was small, including only a few voxels corresponding to the parieto-occipito-temporo-pontine (cortico-reticular) pathway for the contralateral representations of both FCR and ECU, and some of the voxels of FCR activation in the ipsilateral medulla corresponding with the medial lemniscus.

Via visual comparisons with the anatomical brainstem atlases^45,46^, we identified that the largest cluster of FCR activation in the medulla is likely in the ipsilateral ventral medullary reticular nucleus, while the largest cluster of ECU activation is contralateral to the stimulated arm, and in a lateral portion of the pontine nucleus. In two areas, activation of FCR and ECU seemed to overlap; these areas were the contralateral reticular nucleus in the dorsal part of the pons, and in a bilateral dorsal region in the midbrain, likely in correspondence of the periaqueductal gray substance and the raphe nuclei.

**Figure 6:**
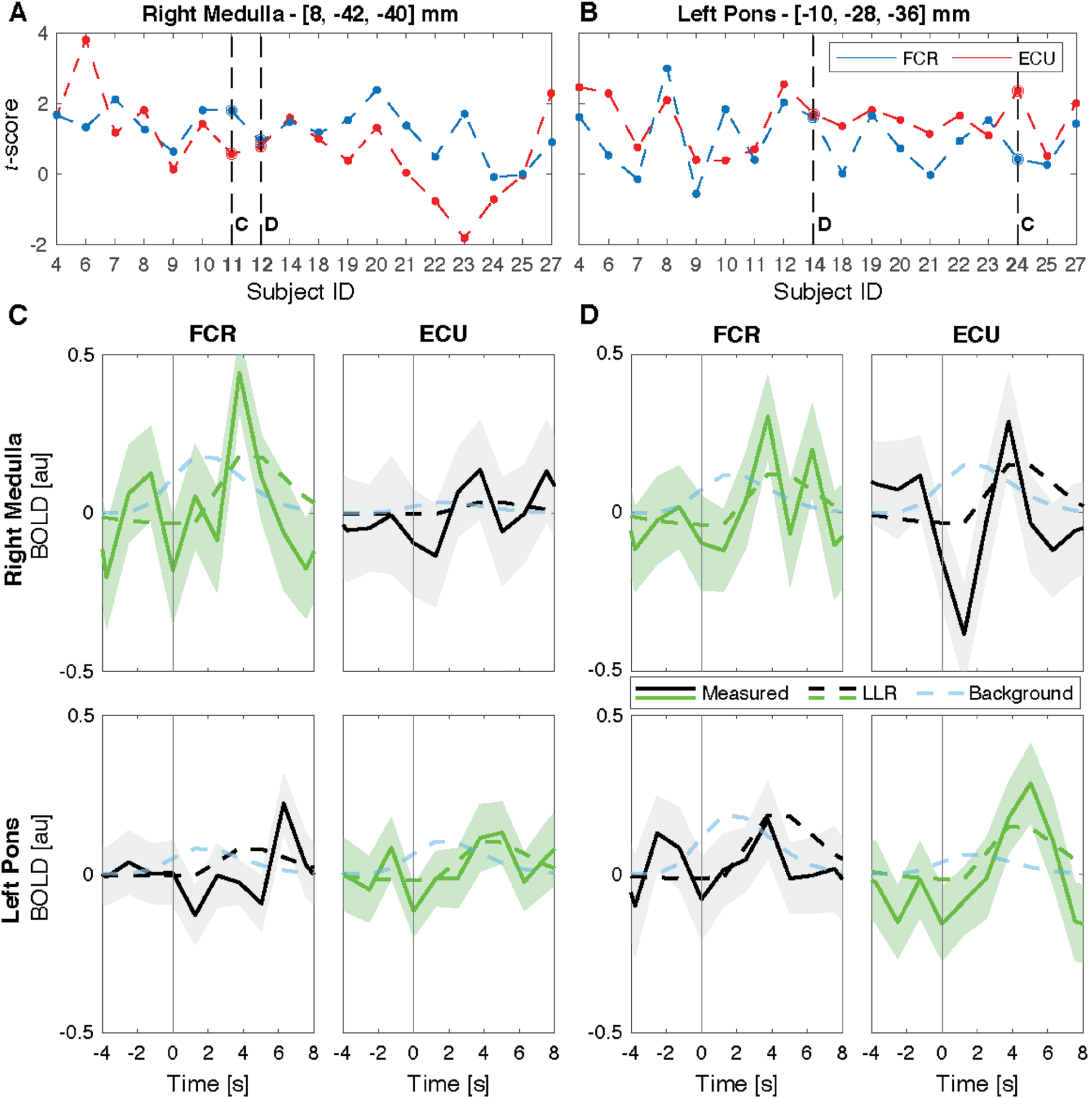
(**A**,**B**) Participant-specific *t* scores obtained for the FCR and ECU activation in the two most significant clusters at the group level in the right medulla (**A**) and left pons (**B**). Dashed lines indicate the subjects that have been selected for the timeseries analysis. Specifically, the participants labeled with (**C**) showed the greatest difference between the statistical scores specific to the FCR and ECU regressors, while the participants labeled with (**D**) showed similar *t* score for the two regressors. (**C**,**D**) Average BOLD signal measured in response to muscle stretch in the selected voxels, for the respective participants, as marked in (**A**) and (**B**). In all graphs, the solid line shows the average residual BOLD response after variance associated to nuisance regressors is removed, while the shaded area represents the 95% confidence interval of the mean response. Dashed lines show the average modeled response respectively for the LLR-specific regressors (green or black line), and for the background-specific regressor (light blue line), in each respective participant. Modeled response for the LLR-specific regressor was scaled based on the appropriate $\beta$ coefficient estimated by the GLM model, while modeled background response, which is not included in the primary GLM, was arbitrarily scaled to match the same amplitude of the LLR-specific regressor. Measured and LLR-specific signals are then color-coded to distinguish the response that is significant at the group level for that specific muscle (green), from the one that is not significant (black).

Via subject-specific analysis of BOLD signal measured from the two clusters with highest statistical scores at the group level, we confirmed the presence of LLR-related BOLD signal specific to FCR stretch in the right medulla and ECU stretch in the left pons (Fig. 6), and that most participants had statistically significant activation in at least one of the voxels of activation identified in the group-level analysis (15/18 or 83.3% for FCR and 14/18 or 77.7% for ECU—Fig. S13).

The subject-specific analysis conducted with the two models suggests that, at least for the clusters of strongest activation at the group level, the dynamics of the measured BOLD signal is more closely associated with LLR dynamics, rather than background activation (Fig. S13).

#### Whole-brain analysis

Muscle-specific activation maps included several clusters of activation spanning cortical, subcortical, and cerebellar regions. Specifically, LLR-related cortical activity specific to FCR included contralateral activation in the sensorimotor cortex—in Brodmann areas (BA) 1, 4a, and 6—, in the insular cortex (Insula Id1), in the inferior parietal lobule (PFop), and in the opercular cortex (Parietal Operculum OP3), and ipsilateral activation in the intra-parietal Sulcus (hIP2). Additionally, bilateral activation was observed in the inferior parietal lobule (PFcm, PFm on the right hemisphere and PFop in the left hemisphere), and in the visual cortex (bilateral BA17 and right BA18) (Fig. 5 bottom and Table 3). Cerebellar activity included contralateral activation in regions I–IV, VI, and Crus I, and ipsilateral activation in regions V, VI, and VIIIa. Finally, activation could also be observed bilaterally in the thalamus, in the putamen, in the right caudate, and in the right medulla (Fig. 5 bottom and Table 3).

Similarly, LLR-related cortical activity specific to ECU included contralateral activation in the sensorimotor cortex (BA1, BA4a, and BA6), in the insular cortex (Insula Id1), and in the opercular cortex, and ipsilateral activity in the inferior parietal lobule (PFm), and in the Broca’s Area (BA44) (Fig. 5 bottom and Table 4). Cerebellar activity included contralateral activation in regions I-VI, VIII, and Crus I, and ipsilateral activation in regions I-VI, and VIII. Finally, activation could also be observed in the left thalamus and putamen, and in bilaterally in the caudate (Fig. 5 bottom and Table 4).

**Table 3:** Whole-brain analysis—FCR-specific activation.

Region		Peak-level		Cluster-level		Peak coordinate [mm]		
Area	Laterality	$\text {t}_{{max}}$	$\text {p}_{\text {FWE}}$	Voxels in cluster	$p_{\text {FWE}}$	x	y	z
**Cortical regions**								
Visual cortex	R	14.94	< 0.001	2294$^c$	< 0.001	10	− 90	10
	R	8.00	0.018	16	0.001	22	− 88	26
	L	7.80	0.024	11	0.003	− 10	− 94	6
Insular cortex	L	12.19	< 0.001	-Part of cluster *f*-		− 38	− 10	− 4
Sensorimotor cortex	L	11.69	< 0.001	467$^a$	< 0.001	− 32	− 38	72
	L	10.73	0.001	-Part of cluster *a*-		− 22	− 38	66
	L	10.64	0.001	-Part of cluster *a*-		− 24	− 30	76
Lingual gyrus	R	11.38	< 0.001	-Part of cluster *c*-		2	− 78	− 6
Inferior parietal lobule	R	8.50	0.009	69$^b$	< 0.001	54	− 36	34
	R	7.75	0.025	-Part of cluster *b*-		56	− 30	28
	L	8.03	0.018	13	0.002	− 62	− 26	30
Intra-parietal sulcus	R	7.47	0.037	-Part of cluster *b*-		44	− 40	34
Operculum	L	8.15	0.015	37	< 0.001	− 40	− 14	16
Temporal gyrus	R	8.30	0.012	11	0.003	50	0	− 44
**Cerebellar regions**								
CB V	R	13.35	< 0.001	-Part of cluster *c*-		2	− 60	− 6
CB VI	R	12.06	< 0.001	103	< 0.001	26	− 72	− 22
	L	9.43	0.003	-Part of cluster *d*-		− 20	− 52	− 28
	L	9.05	0.005	-Part of cluster *d*-		− 32	− 44	− 34
CB I–IV	L	10.31	0.001	65	< 0.001	− 16	− 38	− 30
Crus I	L	9.48	0.003	791$^d$	< 0.001	− 30	− 60	− 36
CB VIIIa	R	8.22	0.014	26	< 0.001	22	− 50	− 50
**Subcortical regions**								
Putamen/thalamus	L	13.13	< 0.001	603$^f$	< 0.001	− 24	− 16	10
Thalamus	R	9.73	0.002	111	< 0.001	22	− 28	6
Caudate	R	9.13	0.004	100$^e$	< 0.001	18	8	14
Putamen	R	8.15	0.015	-Part of cluster *e*-		24	− 2	14
**Brainstem regions**								
Medulla	R	10.68	< 0.001	32	< 0.001	6	− 36	− 38

Superscripts on the cluster volume, when present, refer to clusters with multiple peaks. Coordinates are expressed in MNI space.

**Table 4:** Whole-brain analysis—ECU-specific activation.

Region		Peak-level		Cluster-level		Peak coordinate [mm]		
Area	Laterality	$\text {t}_{{max}}$	$\text {p}_{\text {FWE}}$	Voxels in cluster	$p_{\text {FWE}}$	x	y	z
**Cortical regions**								
Sensorimotor cortex	L	11.80	< 0.001	226$^a$	< 0.001	− 28	− 40	70
	L	9.19	0.003	-Part of cluster *a*-		− 24	− 30	76
	L	8.88	0.005	-Part of cluster *a*-		− 30	− 30	68
	L	8.79	0.005	44$^b$	< 0.001	− 46	− 36	60
	L	7.51	0.028	-Part of cluster *b*-		− 54	− 30	54
Operculum	L	10.83	< 0.001	65	< 0.001	− 38	6	10
Insular cortex	L	8.88	0.005	98$^c$	< 0.001	− 46	8	− 4
	L	8.27	0.010	-Part of cluster *c*-		− 42	− 4	− 4
Inferior parietal lobule	R	8.56	0.007	59	< 0.001	54	− 40	34
Broca’s area	R	7.86	0.018	33	< 0.001	58	12	− 2
**Cerebellar regions**								
CB V	R	15.54	< 0.001	1298$^d$	< 0.001	4	− 66	− 12
	R	10.71	0.001	-Part of cluster *d*-		16	− 54	− 18
CB VI	R	11.12	< 0.001	-Part of cluster *d*-		24	− 70	− 22
	L	11.66	< 0.001	1000$^e$	< 0.001	− 26	− 62	− 20
Cerebellar VIIIa-b	L	11.61	< 0.001	-Part of cluster *e*-		− 26	− 42	− 48
Cerebellar VIIIa	R	9.84	0.001	125$^f$	< 0.001	18	− 44	− 52
Crus I	R	8.05	0.014	23	0.001	46	− 60	− 28
CB I–IV	R-L	7.56	0.026	11	0.005	0	− 48	− 4
	L	8.34	0.009	33	< 0.001	− 12	− 32	− 24
Cerebellar VIIIb	R	7.21	0.043	-Part of cluster *f*-		16	− 56	− 48
**Subcortical regions**								
Thalamus/putamen	L	12.70	< 0.001	356$^g$	< 0.001	− 20	− 14	18
Caudate	R	11.16	< 0.001	208	< 0.001	18	− 6	18
	L	10.19	0.001	-Part of cluster *g*-		− 18	2	18
	L	8.16	0.012	11	0.005	− 4	− 4	10

Superscripts on the cluster volume, when present, refer to clusters with multiple peaks. Coordinates are expressed in MNI space.

## Discussion

In this study, we present and validate StretchfMRI, a novel non-invasive technique that enables direct measurement of the subcortical substrates involved in Long-Latency Responses (LLRs) for the first time in humans. StretchfMRI makes a synergistic use of three key components. First, a custom-made MR-compatible wrist robot (the MR-StretchWrist) is used to apply velocity-controlled perturbations at the wrist joint in order to condition LLRs in the wrist muscles. Second, a whole-brain echo-planar fMRI sequence, modified to include a 225 ms silent window after every acquisition volume, is used to obtain gradient artifact-free recordings of the stretch-evoked EMG activity. Finally, a custom EMG electrode set is used in combination with a learning filter to remove movement artifacts from the stretch-evoked EMG activity, thus allowing measurement of reliable EMG data during fMRI scanning. The key new capability afforded by the developed method is the quantification of stretch-evoked muscle responses induced by robotic perturbations using EMG together with fMRI to quantify corresponding neural responses. This capability was crucial for establishing the somatotopic arrangement of brainstem activity associated to LLRs of flexors and extensors for the first time non-invasively in humans.

### Measurement of stretch-evoked muscle responses during fMRI

The use of reference electrodes has been proposed before for processing noisy EEG signals corrupted from noise deriving from either MRI^30^ or motion artifacts induced during walking^47^. However, the sole use of reference electrodes to filter EMG measured during stretch-evoked muscle responses during MRI afforded a test-retest reliability smaller than the one of the physiological process under study (Figs. 2, 3, S11, S12, Tabs. S8, S9, S10, S11). Instead, in our study, we demonstrate that the combination of an MRI-compatible robot to condition LLR responses with Adaptive Noise Cancellation to filter EMG signals with a new electrode set allowed quantification of LLR responses that did not statistically differ from those measured outside the MRI scanner (Figs. 2, 3, S11, S12, Tabs. S8, S9, S10, S11). Moreover, while sEMG has been used during fMRI for isometric tasks^48^ and ocular movements^49^, we demonstrate for the first time reliable sEMG measurements during fMRI for a dynamic task, and quantify their reliability relative to a highly stereotypical physiological process such as Long-Latency Responses.

We combined two analyses to establish the reliability of StretchfMRI in quantifying LLR amplitudes during MRI. We first used a standard method used for test-retest analysis^35^, to quantify bias and limits of agreement of the mean LLR responses averaged across multiple repetitions for each set of conditions, and paired each value with the respective average measurement obtained outside the MRI. Moreover, to isolate the effects of measurement error from those of physiological variability, we defined our outcome measures as contrasts to reference values of reliability obtained when applying the same statistical methods to measurements obtained in two reference sessions, both performed outside the scanner. The results established that our novel filtering pipeline, which uses adaptive noise cancellation to process data recorded during fMRI, grants an agreement with measurements taken outside MRI that is not statistically different from the agreement obtained by repeating the same experiment twice outside MRI (Figs. 3A,B, S12A,B, Tables S8, S10). Test-retest reliability obtained using alternative processing methods was instead significantly lower than the one obtained for two repeated experiments outside MRI (Figs. 2A,B, S12A,B, Tables S8, S10).

Because it is important to determine the accuracy in quantifying the LLR for each given perturbation during MRI, we conducted an additional analysis that uses measurements from each specific perturbation. With this analysis, we sought to quantify the deviation between each stretch-evoked response during MRI and the distribution of responses measured in a matched reference condition outside the scanner for each subject. Once again, results demonstrate that only the ANC-based processing pipeline allowed us to achieve, during MRI, a variance of stretch-evoked responses for muscles under stretch (Fig. 3C) that is not different from the one obtained outside MRI (Tables S9, S11 and Figs. 3C, S12C).

Overall, these results demonstrate the validity of measurements of LLR amplitude of stretched muscles obtained during fMRI, which allowed us to seek an association between muscle responses and neural function encoded in the BOLD signal.

### Neural correlates of LLRs

The brainstem-specific analysis showed that BOLD signal in the brainstem is modulated by the LLR events in both flexors and extensors. Although previous studies advanced the hypothesis that the brainstem networks should be primarily involved the generation of stretch-evoked responses when task-dependent goals are included in the perturbation protocols^20^, we provide here evidence of stretch-related brainstem activation even in absence of explicit goals (participants were asked to yield to the perturbations). Our results hold true even when using a conservative threshold for quantifying significance of the statistical parametric maps associated with the measured BOLD signal, as the threshold t-statistic obtained with the FWE correction we used is very close to the one resulting from the conservative Bonferroni correction ($t_{FWE}=5.6$ vs. $t_{Bon}=5.8$).

Overall, the muscle-specific statistical parametric maps showed overlapped clusters of activity in the bilateral midbrain and in the contralateral pons, and distinct clusters of activity in the pons and medulla (Fig. 5 and Table 2). Specifically, LLR-related activity for FCR included activation in the right superior medulla and bilaterally in the pons. LLR-related activity for ECU included activation only in the left pons. The laterality of observed pontomedullary arrangement is in partial agreement with the currently accepted double reciprocal model of motor functions in the reticulospinal tract^10,13^, with the neural activity of flexors and extensors expected in the right medullary and left pontine reticular formation, respectively. However, the double reciprocal model does not solely describe the complete pattern of activation observed in the brainstem. Clusters of activation observed bilaterally in the pons for FCR are not explained by the model, which suggests that the double reciprocal model is probably not to be intended in an exclusive manner.

Our measurements of brainstem function also include midbrain activity for both FCR and ECU, which is in agreement with previous studies. Taylor et al.^50^ observed how stimulation of the midbrain can modulate the response of the afferent spindle fibers in response to muscle stretch, thus modulating the stretch-reflex threshold^51^. Additionally, the midbrain, together with other subcortical structures such as thalamus, putamen, caudate nucleus, and basal ganglia, is known to play a crucial role in movement initiation^52^.

The whole-brain exploratory analysis supports the involvement of the cortico-thalamo-cerebellar network for both FCR and ECU. Overlapped clusters of activation can be observed contralaterally in the primary somatosensory cortex, primary motor cortex, and premotor cortex and bilaterally in the thalamus and cerebellum. This observation is in line with our knowledge on the neural basis of motor control^53,54^ and is in agreement with the findings previously obtained on neural substrates of LLRs^20^. Additional activation could also be observed in secondary somatosensory areas—i.e. left parietal operculum, left insular cortex—for both FCR and ECU. Both areas are part of the somatosensory system and have been observed to be involved in processing tactile sensation and attention^55^, and the sense of bodily-ownership and proprioception^56^. Finally, overlapped clusters of activation referring to the two muscles can be observed in the right caudate nucleus, left putamen, and the right inferior parietal lobule. While the involvement of the inferior parietal lobule in controlling motor actions is still under debate, with few studies that propose its involvement in coding different motor actions^57^, the role of putamen and caudate nuclei is more established^52,58^. Both subcortical regions have been observed to be involved in postural maintenance and sensory-motor integration^59^ and in controlling movement initiation^52^.

fMRI activation data obtained using StretchfMRI afford test-retest reliability that varies according to different brain regions. In terms of a standard reliability scale^60^ used in other fMRI test-retest reliability studies^38,39,61^, we observed moderate to good reliability in the whole brain as well as for all cortical and cerebellar ROIs both at the subject and the group level (Table S12 and Fig. 4). On the other hand, we observed lower reliability in subcortical ROIs, with poor overlap and poor to fair ICC (Table S12 and Fig. 4). While the reliability for the brainstem is low, it is important to consider two main factors. First, event-related designs, which are necessary to associate stretch-evoked muscle activity with BOLD signal, are known to have a lower test-retest reliability compared to a standard block design^62^. Second, the smaller FWHM of the smoothing filter used for the brainstem-specific analysis negatively affected the test-retest reliability metrics^38^. As a comparison, the $\text {ICC}_g$ score quantified for the brainstem changed from 0.28 to 0.43 and 0.24 to 0.39 for FCR and ECU, respectively, when using a smoothing filter with an 8-mm FWHM. As such, low test-retest reliability presented here is obtained in light of increasing sensitivity to capture small regional variations of nuclei function. To the best of our knowledge, this is the first study quantifying motor function in the brainstem using event-related protocols, and reporting values of test–retest reliability for their protocol. Nonetheless, in all ROIs the reliability metrics assume values that are comparable with those reported by previous studies for both cortical^38,39^ and subcortical ROIs^61^.

## Study limitations

A possible confound of the experimental design pursued in this paper relates to the association between the states of agonist muscle contraction and ensuing stretch. Because these states always appear in sequence, and are typically only a few seconds apart, it is possible that some clusters of activation might arise, at least in part, from activity associated with background contraction instead of LLR events. We conducted a supplementary analysis to understand the likelihood of this possibility and potentially to rule it out. The results of the supplementary analysis indicate that even after accounting for activation associated with background activity, the LLR-specific activation maps are very similar to the ones obtained in the main analysis. Specifically, statistically significant voxels (Fig. S4 and Table S3), while fewer in number, are generally aligned with those identified by the primary GLM in both muscle-specific activation maps (Fig. 5 and Table 2). Moreover, the evidence that no supra-threshold voxels associated with the background-specific regressors could be identified in the brainstem, together with the results of the simulation analysis done to quantify specificity and sensitivity of the GLMs, suggest that the clusters of activation included in the brainstem are likely to be associated with true LLR-related activity. Finally, qualitative observation of the timeseries measured in the voxels with highest statistical scores at the group level (Fig. 6) suggest that activation in the right medulla and left pons is indeed primarily associated with LLRs and not with background activity.

The study is limited in the spatial specificity achievable in identifying different nuclei within the brainstem. While activations can be localized roughly in terms of their medio-lateral, rostro-caudal, and dorso-ventral coordinates within the pons or medulla, matching the areas of activation with specific brainstem nuclei is very difficult due to their small size compared to the 2mm resolution of the EPI scans. Also, precise localization is hindered by the lack of nuclei-specific atlases of the descending tracts through the brainstem, uncertain orientation of anatomical templates, and by the inaccuracy of alignment of group-level data. While the main clusters of activation appeared to localize with the largest regions that are undifferentiated in the atlases (i.e., pontine nucleus, medullary reticular formation, periaqueductal gray substance), we can not currently rule out the involvement of smaller nuclei such as the raphe nuclei or the olivary nuclei in the measured activation. In future work, we plan to follow-up our whole-brain sequence with higher resolution, small FOV sequences directly targeted at the brainstem, to gain more insight on the involvement of different groups of nuclei in the motor responses. Moreover, it is possible that some of the activations in the periaqueductal region may be driven by noise from surrounding regions, not completely removed by the operation of masking, which was applied after smoothing in this paper.

The validation of the signal processing pipeline is based on the contrast between the reliability of a test condition (comparison between IN and OUT1 sessions), and a control condition (comparison between OUT1 and OUT2 sessions). It is possible that the reliability of the control condition is artificially reduced due to the fact that OUT1 and OUT2 sessions are conducted more than one hour apart. As such, we repeated the analysis using the comparison between the first 50% of trials and the last 50% of trials as the control condition, and using as test condition the comparison between the first 50% of trials in the IN1 condition and the first 50% of trials in the OUT1 condition (see Fig. S14:S15 for the results). The results of this alternative analysis are very similar as the original analysis reported in the paper, which indicates that the baseline reliability is not artificially reduced by the time elapsed between conditions, thus validating the reliability of the presented technique.

## Conclusions

StretchfMRI is a novel technique that for the first time enables in-vivo measurements of brainstem function during stretch-evoked responses in humans. Statistical parametric maps of muscle-specific responses in the brainstem in part support established models of the organization of motor function in the pontomedullary reticular formation^10,13^, showing how excitatory stretch-evoked responses in the flexors and extensors correlate with neural activity in the ipsilateral medulla and contralateral pons, respectively. StretchfMRI allows for the first time quantitative analysis of function in a secondary motor pathway, the reticulospinal tract (RST), thus enabling new investigations in both basic and translational neuroscience. As an example, StretchfMRI can be used to further investigate the task-dependent modulation of brainstem activity associated with LLRs. Because function in the RST has been advanced to play a role in post-stroke recovery or impairment^16,17^, but measured indirectly and with limited spatial specificity, it is possible that StretchfMRI will result in an improved understanding of mechanisms of recovery and impairment from CST lesions.

## Supplementary Information

Supplementary Information.
