# Supplementary material for: Measurement of stretch-evoked brainstem function using fMRI
Source: Sci Rep. 2021 Jun 15;11:12544. doi: 10.1038/s41598-021-91605-5 (PMC8206209; doi:10.1038/s41598-021-91605-5)
Supplement: Supplementary file 1 — Supplementary Information. [file 41598_2021_91605_MOESM1_ESM.pdf]

Measurement of stretch-evoked brainstem function using fMRI

Andrea Zonnino[1], Andria J Farrens[1], David Ress[2], and Fabrizio Sergi[1,*]

[1]Human Robotics Laboratory, Department of Biomedical Engineering, University of Delaware, Newark DE, 19713, USA
[2]Department of Neuroscience, Baylor College of Medicine, Houston, TX, 77020, USA
[*]fabs@udel.edu

Supplementary Materials

1 Behavioral Results

Participants were able to achieve cued contraction states for both reference isometric contractions (used for normalization of filtered EMG data), and for muscle pre-conditioning (used to trigger robotic perturbations). The mean background torque measured during the set of reference isometric contractions, applied in flexion and extension prior to the beginning of each perturbation session, was (mean $\pm$ stdev) 482 $\pm$ 21mNm and 486 $\pm$ 19 mNm respectively, for session OUT_1, 486 $\pm$ 29 mNm and 482 $\pm$ 20 for sessions IN_1 and IN_2, 498 $\pm$ 22 mNm and 484 $\pm$ 19 mNm for session OUT_2. The mean filtered EMG amplitude measured during the same isometric contractions in the FCR and ECU was 24.61 $\pm$ 15.18 μV and 91.89 $\pm$ 40.26 μV respectively, for session OUT_1, 36.23 $\pm$ 34.99 μV and 99.57 $\pm$ 48.48 μV for sessions IN_1 and IN_2, and 27.19$\pm$19.64 μV and 90.70 $\pm$ 29.04μV for session OUT_2.

The mean background torque measured prior to each perturbation (average between measurements during flexion and extension) was 183 $\pm$ 19 mNm, 192 $\pm$ 20 mNm, 191 $\pm$ 18 mNm, 187 $\pm$ 18 mNm, respectively for OUT_1, IN_1, IN_2, and OUT_2, with the time required to trigger a robotic perturbation equal to 1.55 $\pm$ 0.68 s, 2.90 $\pm$ 1.75 s, 2.78 $\pm$ 1.55 s, 1.44 $\pm$ 0.62 s, respectively for the four sessions. The mean time elapsed between two consecutive perturbations was 10.12 $\pm$ 1.43 s, 14.08 $\pm$ 3.03 s, 14.11 $\pm$ 2.40 s, 9.88 $\pm$ 1.36 s. The group level distributions of the parameters listed above are shown in the Fig. S1.

The mean timeseries of the measured FE angle and FE torque are reported in Fig S2.

2 Supplementary analysis of fMRI data

The GLM selected to partition the variance of the measured BOLD signal (Eq. 4 of the main manuscript) does not include separate regressors for background activity and LLR amplitude. Because participants are asked to contract their muscles prior to each perturbation, there is an intrinsic statistical association between the muscle state before the perturbation (i.e., background muscle activity) and the perturbation itself (i.e., reflex response). As such, given the poor temporal resolution of fMRI, it is possible that some of the variance in the BOLD signal explained by LLR-related regressors might actually arise from the neural activity associated to background contraction. To rule out this concern, we conducted two analyses, one based on simulated data, and one based on the implementation of a different GLM on the fMRI data collected in Experiment 2.

2.1 Model-based analysis

We initially sought to quantify whether the implemented analysis method is sufficiently sensitive and specific in identifying neural activity related to LLR in presence of correlated background activity. To this goal we have conducted a simulated signal analysis following the methods presented in our previous work[1].

2.1.1 Methods

We simulated nine different representative brain voxels V_1 to V_9 that selectively respond only to linear combinations of conditions of flexor and extensor muscle activity, defined in terms of LLR events ($\mathbf{H}^{\star}_{\mathbf{F}}$ and $\mathbf{H}^{\star}_{\mathbf{E}}$, respectively for flexor and extensor) and background contraction ($\mathbf{B}^{\star}_{\mathbf{F}}$ and $\mathbf{B}^{\star}_{\mathbf{E}}$, respectively for flexor and extensor). Specifically, $\mathbf{H}^{\star}_{\mathbf{F}}$ and $\mathbf{H}^{\star}_{\mathbf{E}}$ are obtained by convolution

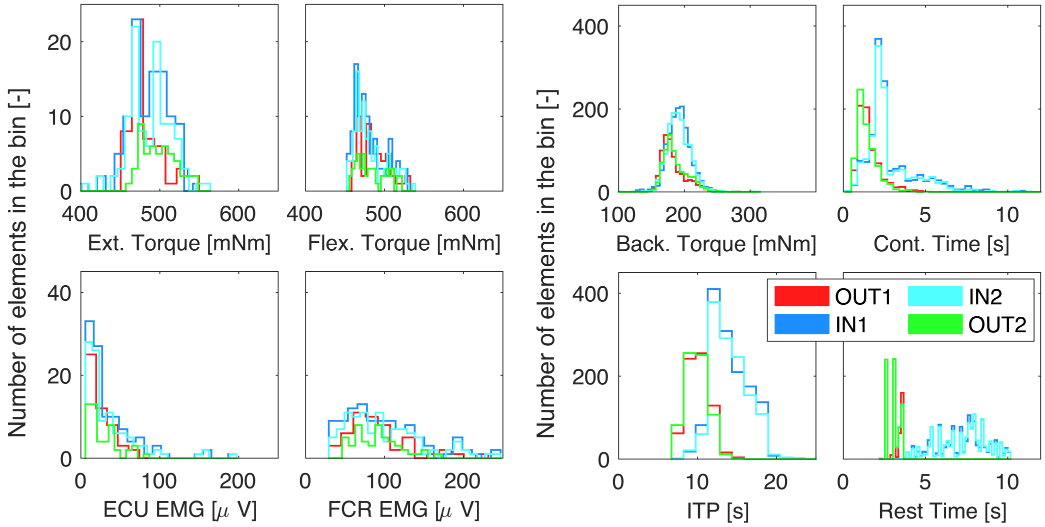

Figure S1. (**A**) Distribution of the behavioral parameters measured during the reference isometric contraction applied prior to the beginning of each session and utilized for the normalization of the stretch-evoked EMG recordings. (**B**) Distribution of the perturbation-specific behavioral parameters.

of rectangular functions (duration: 50 ms, onset: 50 ms after perturbation start, amplitude: $\tilde{\mathbf{H}}_{\mathbf{FCR}}|^{\mathbf{st}}_{\mathbf{ANC,P4}}$ and $\tilde{\mathbf{H}}_{\mathbf{ECU}}|^{\mathbf{st}}_{\mathbf{ANC,P4}}$, respectively) with the standard HRF (Fig. S8). Similarly, the terms $\mathbf{B}^{\star}_{\mathbf{F}}$ and $\mathbf{B}^{\star}_{\mathbf{E}}$ are given by the convolution of rectangular functions (unitary magnitude in the time interval when the measured torque has a magnitude greater than 25% of the desired torque) with the standard HRF (Fig. S8). To simulate all four conditions, we used the EMG and torque data measured in a representative participant (participant P4). A schematic representation of the simulated voxels is reported in Tabs. S1 and S2.

Table S1. Definition of voxels used in the simulation analysis

Effects	Flexor	Extensor	Both
$\mathbf{H}^{\star}$	V_1	V_2	V_3
$\mathbf{B}^{\star}$	V_4	V_5	V_6
$\mathbf{H}^{\star}+\mathbf{B}^{\star}$	V_7	V_8	V_9

For each of the simulated voxels, we then added zero-mean Gaussian noise and down-sampled the signal with a period T_R = 1.225 s, to simulate realistic BOLD measurements specific to either cortical voxels (Signal-To-Noise ratio equal to 1.2) or brainstem voxels (Signal-to-Noise ratio equal to 0.5). Finally, we quantified the sensitivity that the two proposed GLMs (GLM_1 in Eq. 4 and GLM_2 in Eq. S1) have in identifying the four different regressors, by computing the true positive (TPR) and false positive (FPR) rates afforded when either of the two GLM is used to fit the signal simulated in each of the nine different voxels.

2.1.2 Results

The results show that the model GLM_1 affords the highest TPR for LLR-specific voxels in both cortical and brainstem voxels, and for both flexor and extensor regressors (V_1 and V_2, respectively; Fig. S3A, top row and Table S2). However, it also returns high FPR for LLR-related activity in voxels whose activity is associated to the application fo background torque (Fig. S3A, middle and Table S2), though the FPR is smaller in the brainstem voxels than in the cortical ones.

The inclusion of the background-specific regressors drastically reduces the FPR of LLR-related activity in background-specific voxels for both flexor and extensor (V_4 and V_5, respectively; Fig. S3B, middle row and Table S2). However, while TPR remains high in LLR-specific voxels (Fig. S3A, top row and Table S2), it decreases to almost zero in voxels that responds to both LLR and background activity (V_7 and V_8; Fig. S3A, bottom row and Table S2).

2.2 Alternative GLM analysis

We conducted an alternative statistical analysis for the BOLD fMRI data that aims to determine the LLR-related neural activity including a correction for the possible confounding effect introduced by the background-related activity.

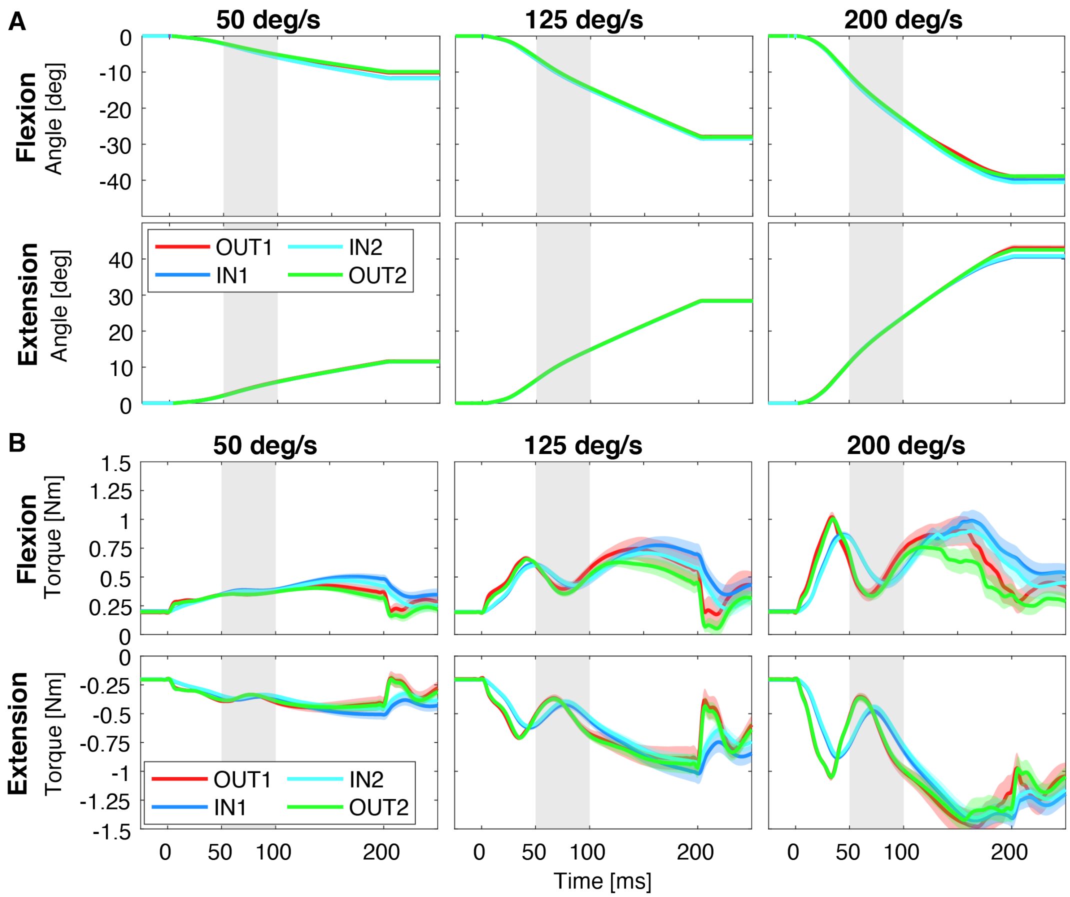

Figure S2. Group-average of the flexion/extension angle (A) and torque (B) measured in response to RaH perturbations applied at different velocities. In all graph the thick line represents the mean and the shaded area the 95% confidence interval. To facilitate interpretation on the graphs a gray shaded area has been included in each graph in the time interval where a LLR is expected.

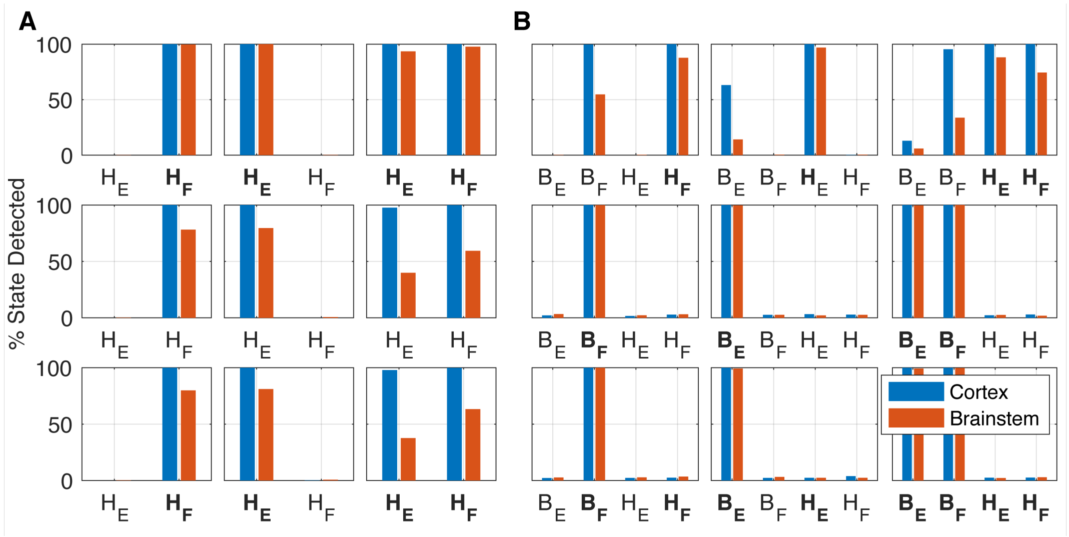

Figure S3. Identification rate for GLM_1 (**A**) and GLM_2 (**B**). Bold labels indicate true positive association between the indicated regressor and the BOLD signal measured in the corresponding voxel.

Table S2. Percent state detected in the different voxels for the two GLMs. Bolded labels represents true positive identification rates, while un-bolded labels represents false positive identification rate

Voxel	Effects	H_F	H_E	B_F	B_E	H_F	H_E	B_F	B_E
**GLM$_1$**		Cortex				Brainstem			
V_1	$\mathbf{H^\star_F}$	**1**	0	-	-	**0.99**	0.01	-	-
V_2	$\mathbf{H^\star_E}$	0	**1**	-	-	0.01	**1**	-	-
V_3	$\mathbf{H^\star_F + H^\star_{ECU}}$	**1**	**1**	-	-	**0.97**	**0.93**	-	-
V_4	$\mathbf{H^\star_E}$	1	0	-	-	0.78	0.01	-	-
V_5	$\mathbf{B^\star_E}$	0	1	-	-	0.01	0.79	-	-
V_6	$\mathbf{B^\star_F + B^\star_{ECU}}$	0.99	0.97	-	-	0.59	0.39	-	-
V_7	$\mathbf{H^\star_F + B^\star_F}$	**1**	0	-	-	**0.79**	0.01	-	-
V_8	$\mathbf{H^\star_E + B^\star_E}$	0.001	**1**	-	-	0.01	**0.81**	-	-
V_9	$\mathbf{H^\star_F + H^\star_E + B^\star_F + B^\star_E}$	**0.99**	**0.97**	-	-	**0.63**	**0.37**	-	-
**GLM$_2$**		Cortex				Brainstem			
V_1	$\mathbf{H^\star_F}$	**1**	0	1	0	**0.88**	0	0.55	0
V_2	$\mathbf{H^\star_E}$	0	**1**	0	0.63	0	**0.97**	0	0.14
V_3	$\mathbf{H^\star_F + H^\star_E}$	**1**	**1**	0.95	0.13	**0.74**	**0.88**	0.34	0.06
V_4	$\mathbf{B^\star_F}$	0.03	0.02	**1**	0.02	0.03	0.02	**1**	0.03
V_5	$\mathbf{B^\star_E}$	0.03	0.03	0.03	**1**	0.03	0.02	0.03	**1**
V_6	$\mathbf{B^\star_F + B^\star_E}$	0.03	0.02	**1**	**1**	0.02	0.03	**1**	**1**
V_7	$\mathbf{H^\star_F + B^\star_F}$	**0.03**	0.02	**1**	0.02	**0.04**	0.03	**1**	0.03
V_8	$\mathbf{H^\star_E + B^\star_E}$	0.04	**0.02**	0.02	**1**	0.02	**0.02**	0.03	**0.99**
V_9	$\mathbf{H^\star_F + H^\star_E + B^\star_F + B^\star_E}$	**0.03**	**0.03**	**1**	**1**	**0.03**	**0.02**	**1**	**0.99**

Bolded number represents true positive identification rates, while un-bolded numbers represents false positive identification rates

2.2.1 Methods

Methods closely followed those described in Sec. 2.5 for most aspects. Separately for each session, we performed two first-level analyses to determine activation in the whole brain and in the brainstem. While we used the same general linear model (GLM) for the whole-brain and brainstem analyses, we used regionally-specific hemodynamic response functions (HRF) for different brain regions. A brainstem-specific HRF[2] was used to assess activation in the brainstem, while a standard HRF was used to assess activation in all other regions. In this alternative GLM (GLM_2) analysis, the neural response was modeled as:

$$y = \beta_{\mathrm{FCR,L}} H^\star_{FCR} + \beta_{\mathrm{ECU,L}} H^\star_{ECU} + \beta_{\mathrm{FCR,B}} B^\star_{FCR} + \beta_{\mathrm{ECU,B}} B^\star_{ECU} + \beta_0 + \boldsymbol{\beta_R} R \quad \text{(S1)}$$

where $H^\star_{FCR}$ and $H^\star_{ECU}$ are obtained by convolution of rectangular functions (duration: 50 ms, onset: 50 ms after perturbation start, amplitude: $\tilde{\mathbf{H}}_{\mathbf{FCR}}|^{\mathbf{st}}_{\mathbf{ANC}}$ and $\tilde{\mathbf{H}}_{\mathbf{ECU}}|^{\mathbf{st}}_{\mathbf{ANC}}$, respectively) with the appropriate HRF (Fig. S8). The terms $B^\star_{FCR}$ and $B^\star_{ECU}$ are now added to the model to account for metabolic activity associated with muscle-specific pre-perturbation (i.e., background) contraction state. They are similarly obtained convolving rectangular functions (unitary magnitude in the time interval when the measured torque is in magnitude greater than 25% of the desired torque) with the appropriate HRF (Fig. S8). As in all analyses, a set of 6 nuisance regressors **R** was included to account for variance in the measured signal associated with the 3D head movements (translation and rotations).

2.2.2 Results

For the brainstem-specific analysis, muscle-specific maps included several clusters of activation associated only to the LLR-specific regressors (Fig. S4, Table S3). In fact, no supra-threshold voxels were identified to correlate with the background-specific regressors (Fig. S5). LLR-related activity specific to FCR included a total of 18 voxels, including two clusters spanning bilaterally the midbrain, two clusters spanning bilaterally the pons, and two clusters in the right superior medulla (Fig. S4 top, and Table S3). LLR-related activity specific to ECU included a total of 16 voxels, including one voxel in the right midbrain, four clusters spanning bilaterally the pons, and one cluster in the right medulla (Fig. S4 top, and Table S3).

In contrast to what we observed in the brainstem-specific analysis, in the whole brain analysis muscle-specific maps included several clusters of activation associated to both LLR-specific (Fig. S4 bottom, Table S4, and Table S5) and background-specific regressors (Fig. S4 bottom, Table S6, and Table S7). Specifically, cortical activation associated to the two background-specific regressors ($\mathbf{B^\star_{FCR}}$ and $\mathbf{B^\star_{ECU}}$) primarily included overlapped clusters of activation in the contralateral sensorimotor cortex and

inferior parietal lobule, in the ipsilateral insular and opercular cortex, and in the bilateral visual cortex. Cerebellar activity included overlapped clusters in the ipsilateral region V and contralateral region VI, while in the subcortical regions activity was primarily detected in the contralateral putamen (Fig. S4 bottom, Table S6, and Table S7). LLR-related cortical activity specific to FCR primarily included contralateral activation in the sensorimotor cortex, inferior parietal lobule and bilateral activation in the visual cortex. Cerebellar activity could be instead observed bilaterally in the regions I-IV and VIII, and contralaterally in the region VI and in the Crus II, while subcortical activity could be observed in the contralateral putamen and thalamus and in the ipsilateral caudate nucleus (Fig. S4 bottom, Table S4). As for the LLR-related activity specific to ECU, no subcortical activity could be observed, with cortical activity observable only in the ipsilateral visual cortex. Cerebellar activity was instead more diffuse and included bilateral activation in the region VIII and Crus I, ipsilateral activity in the regions V and X, and contralateral activity in the region VI and in the Crus II (Fig. S4 bottom, Table S4).

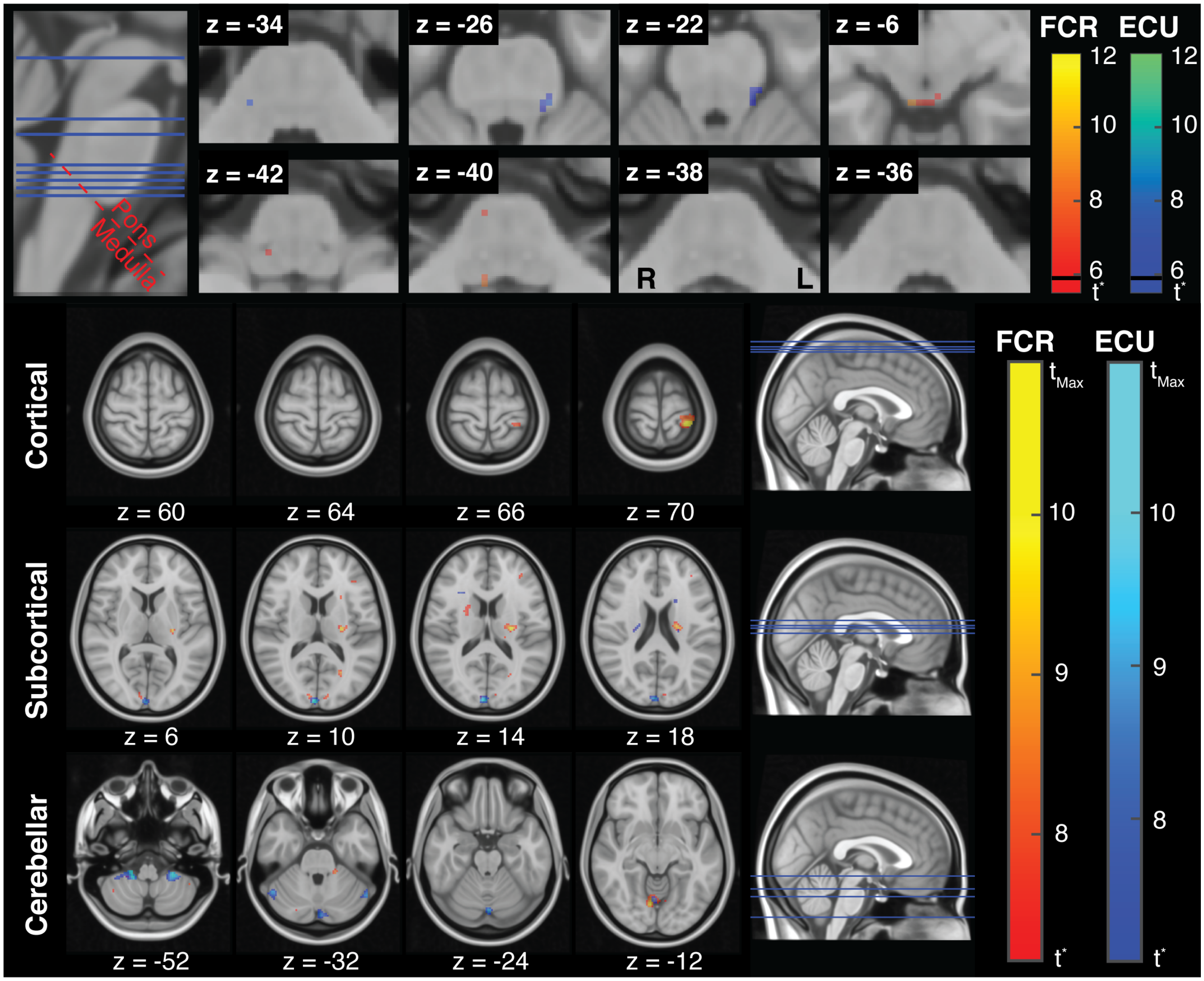

Figure S4. (**Top**) LLR-specific activation maps in the brainstem for FCR and ECU. The maps refer to contrasts $\beta_{FCR,L} > 0$ and $\beta_{ECU,L} > 0$ obtained for the brainstem-specific analysis. The threshold t-statistic, obtained after FWE correction, was equal to 5.62 for both regressors. For reference, the t-statistic that refer to a Bonferroni correction (t_{Bon}= 5.8) is marked on each colorbar with a black line. (**Bottom**) LLR-specific activation maps in the whole-brain for FCR and ECU. The threshold t-statistic, obtained after FWE correction, was set to 7.28 and 6.99, respectively for FCR and ECU, while t_{Max} was equal to 12.81 and 11.95, respectively. Colorbars are saturated at t = 10 for better visualization of t-statistic gradients. All statistical parametric maps are overlaid on axial slices of the standard Montreal Neurological Institute 152 template, with reported z coordinate in mm

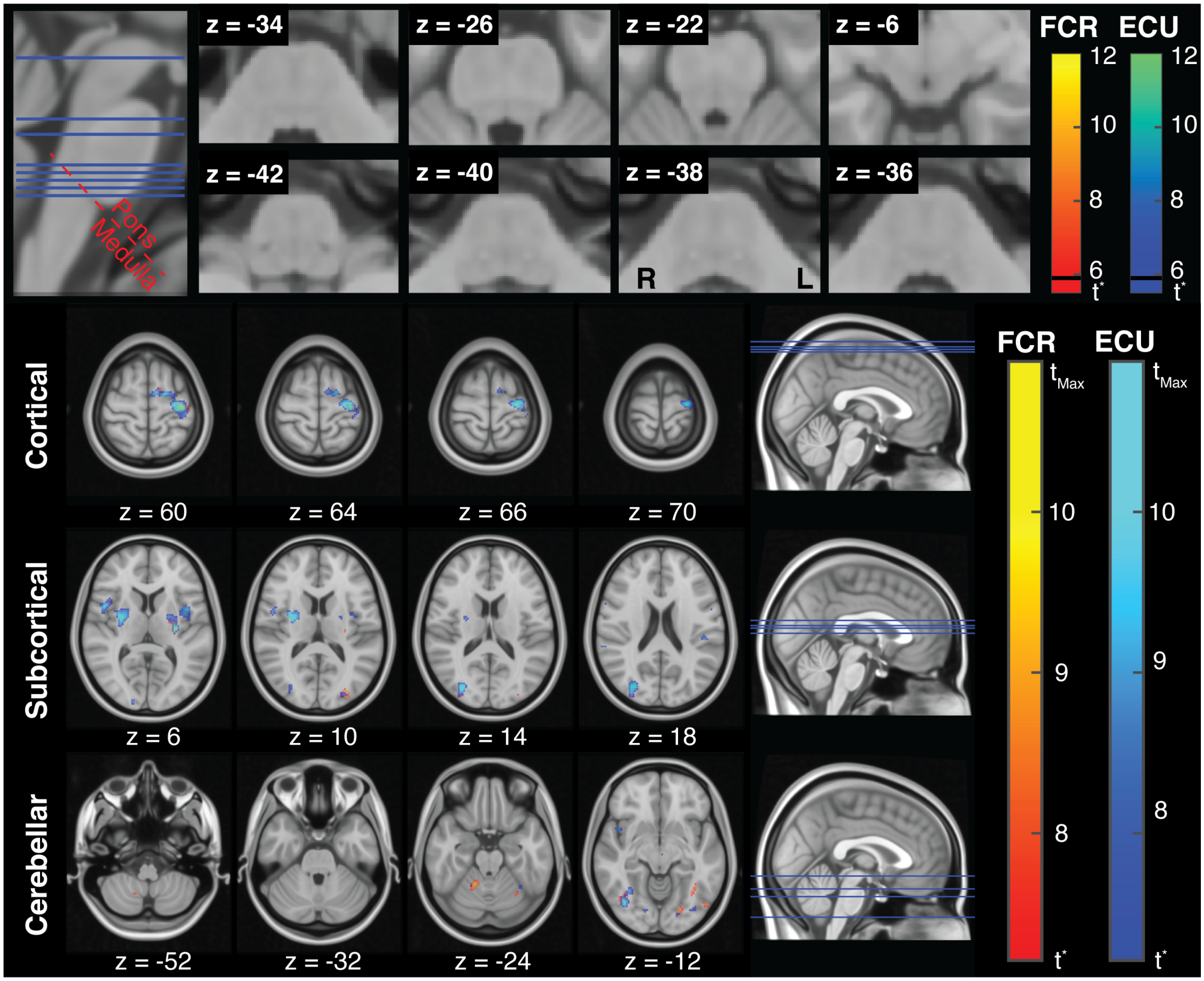

Figure S5. (**Top**) Activation maps in the brainstem for FCR and ECU background activity. The maps refer to contrasts $\beta_{\text{FCR,B}} > 0$ and $\beta_{\text{ECU,B}} > 0$ obtained for the brainstem-specific analysis. The threshold t-statistic, obtained after FWE correction, was equal to 5.62 for both regressors. For reference, the t-statistic that refer to a Bonferroni correction (t_{Bon}= 5.8) is marked on each colorbar with a black line. (**Bottom**) Activation maps in the whole brain for FCR and ECU background activity. The threshold t-statistic, obtained after FWE correction, was set to 7.27 and 7.17, respectively for FCR and ECU, while t_{Max} was equal to 11.44 and 16.66, respectively. Colorbars are saturated at t = 10 for better visualization of t-statistic gradients. All statistical parametric maps are overlaid on axial slices of the standard Montreal Neurological Institute 152 template, with reported z coordinate in mm

2.3 Discussion

The results of the simulation analysis show that voxels that are positively associated with the regressor $\mathbf{H}^{\star}_{\mathbf{F}}$ in GLM_1 might respond to LLR activity, background activity, or both–i.e. V_1, V_4, or V_7, respectively (Fig. S3A, first column). On the contrary, when using GLM_2, voxels that are positively associated with the regressor $\mathbf{H}^{\star}_{\mathbf{F}}$ can only respond to LLR-related activity–i.e. V_1–since a 0% positive identification rate characterize voxels V_4 and V_7. For all three voxels, however, the background-specific regressor $\mathbf{B}^{\star}_{\mathbf{F}}$ dominates, reducing the specificity of the regressor $\mathbf{B}^{\star}_{\mathbf{F}}$ in voxels that only respond to LLR activity (V_1) and the sensitivity of the regressor $\mathbf{H}^{\star}_{\mathbf{F}}$ in voxels that instead respond to both LLR and background activity (V_7), for both cortical and brainstem voxels.

The same observation can be made considering voxels that respond to activity in the extensors. Therefore, it is possible to conclude that when using the model GLM_2, background related activity will always be identified if present, and dominate over

the LLR-related regressor. However, the brainstem activation maps obtained using the GLM_2 model show the complete absence of voxels whose signal is significantly associated with the background specific regressors (Fig. S5). This evidence, together with the observation that the clusters of activations included in the LLR-specific regressors (Fig. S4, Table S3) are closely aligned with the clusters of activations identified by GLM_1 (Fig. 3, Table 1), suggest that the identified brainstem activity is truly associated with LLR-related activity and not with background activation. The insight from the two analyses presented above advances that it is unlikely that some of the variance in the BOLD signal explained by LLR-related regressors might arise from the neural activity associated to background contraction.

In contrast to the similarity observed between the results obtained for the two GLMs in the brainstem-specific analysis, at the whole brain level the two GLMs produce fairly different results. Since both LLRs and isometric contractions are intrinsic motor actions, they are expected to share most of the same cortical substrates. As such, as predicted by the model, it is possible to observe a decrease of the sensitivity that the model has in identifying LLR-related activity, mostly evident in ECU-specific maps (Fig. S4 bottom, Table S4).

In summary, due to the co-linearity between the LLR-specific and background-specific regressors–i.e. after the application of a background torque there is always a perturbation that elicits a reflex response in the pre-conditioned muscles–our experimental protocol does not afford enough statistical power to reliably decouple the LLR-related and background-related activity at the cortical level. However, as demonstrated by the combination of the results of the simulation analysis and of the alternative GLM analysis, it is very likely that the measured activations are specific to LLR-related responses, and minimally affected by statistically associated activity arising from background contraction.

Table S3. Brainstem activation – alternative GLM analysis

**Brainstem Region**		**Muscle**	**Peak-Level**		**Cluster-Level**		**Peak coordinate** [mm]		
Area	Laterality		t_{max}	p_{FWE}	Voxels in cluster	p_{FWE}	x	y	z
Midbrain	R	FCR	8.86	<0.001	11	<0.001	6	-36	-6
	L	FCR	5.84	0.02	1	0.032	-4	-38	-16
	R	ECU	6.13	0.018	1	0.018	8	-32	–16
Pons	L	FCR	6.77	0.005	2	0.007	-10	-26	-32
	R	FCR	5.97	0.024	1	0.018	8	-22	-40
	R	ECU	7.20	0.002	8	<0.001	16	-30	-32
	L	ECU	7.20	0.002	11[a]	<0.001	-12	-32	-26
	L	ECU	6.57	0.008	2	0.008	-8	-34	-44
	L	ECU	6.21	0.015	- part of Cluster *a* -		-10	-32	-22
Medulla	R	FCR	8.09	<0.001	2	0.007	8	-44	-40
	R	FCR	5.64	0.047	1	0.018	10	-36	-42
	R	ECU	6.10	0.019	2	0.008	8	-40	-50

Superscripts on the cluster volume, when present, refer to clusters with multiple peaks. Coordinates are express in MNI space.

Table S4. LLR-related activation for FCR in the whole brain – alternative GLM analysis

**Region**		**Peak-Level**		**Cluster-Level**		**Peak coordinate** [mm]		
Area	Laterality	t_{max}	p_{FWE}	Voxels in cluster	p_{FWE}	x	y	z
**Cortical Regions**								
Sensorimotor Cortex	L	12.82	<0.001	135[a]	<0.001	-32	-38	72
	L	9.98	0.002	- part of Cluster *a* -		-24	-30	76
Lingual Gyrus	R	10.77	0.001	377[b]	<0.001	8	-70	-8
Visual Cortex	R	9.18	0.004	- part of Cluster *b* -		8	-92	12
	R	9.12	0.005	- part of Cluster *b* -		10	-84	4
	L	8.14	0.016	35[c]	<0.001	-4	-90	24
	R	8.06	0.018	- part of Cluster *d* -		8	-88	28
	L	7.90	0.022	- part of Cluster *c* -		-10	-90	12
Inferior parietal lobule	L	9.55	0.003	68	<0.001	-44	-52	38
Frontal Pole	R	8.64	0.008	10	0.003	40	38	4
	L	8.21	0.014	10	0.003	-36	40	18
Paracingulate Gyrus	L	8.39	0.011	20	0.001	-8	34	32
Cuneal Cortex	R	8.39	0.011	75[d]	<0.001	10	-82	38
**Cerebellar Regions**								
CB I-IV	R	9.97	0.002	105	<0.001	26	-32	-36
	L	8.51	0.010	- part of Cluster *e* -		-18	-36	-30
CB VI	L	9.10	0.005	117[e]	<0.001	-24	-38	-36
CB VIII	L	9.05	0.005	- part of Cluster *e* -		-30	-44	-42
	R	8.37	0.012	65	<0.001	36	-56	-40
	R	8.23	0.014	14	0.002	42	-64	-36
Crus II	L	8.00	0.019	23	<0.001	-32	-60	-40
**Subcortical Regions**								
Putamen/Thalamus	L	10.91	<0.001	115[f]	<0.001	-24	-16	16
	L	9.57	0.003	- part of Cluster *f* -		-26	-18	8
Caudate	R	8.04	0.018	26	<0.001	24	0	18

Superscripts on the cluster volume, when present, refer to clusters with multiple peaks. Coordinates are expressed in MNI space.

Table S5. LLR-related activation for ECU in the whole brain – alternative GLM analysis

**Region**		**Peak-Level**		**Cluster-Level**		**Peak coordinate** [mm]		
Area	Laterality	t_{max}	p_{FWE}	Voxels in cluster	p_{FWE}	x	y	z
**Cortical Regions**								
Visual Cortex	R	10.61	0.001	139[a]	<0.001	6	-94	14
	R	8.01	0.012	- part of Cluster *a* -		0	-88	30
**Cerebellar Regions**								
CB X	R	11.96	<0.001	81[b]	<0.001	20	-36	-50
CBVIII	R	11.74	<0.001	- part of Cluster *b* -		16	-44	-52
	L	10.20	0.001	142[c]	<0.001	-26	-40	-50
	R	7.71	0.019	- part of Cluster *b* -		34	-42	-50
	L	7.39	0.029	- part of Cluster *c* -		-18	-48	-46
	L	7.21	0.037	- part of Cluster *c* -		-14	-44	-54
Crus I	L	9.58	0.002	71	<0.001	-52	-52	-34
	R	9.38	0.002	92[d]	<0.001	50	-58	-32
	L	8.86	0.004	- part of Cluster *d* -		-52	-62	-30
	R	8.14	0.010	15	0.004	38	-44	-40
Crus II	L	9.06	0.003	225[e]	<0.001	-2	-84	-32
Vermis IV	L	9.04	0.003	- part of Cluster *e* -		-2	-80	-24
CB V	R	8.87	0.004	- part of Cluster *e* -		4	-66	-8
CB VI	L	8.16	0.010	19	0.003	-40	-40	-36

Superscripts on the cluster volume, when present, refer to clusters with multiple peaks. Coordinates are expressed in MNI space.

Table S6. Background activation for FCR in the whole brain – alternative GLM analysis

**Region**		**Peak-Level**		**Cluster-Level**		**Peak coordinate** [mm]		
Area	Laterality	t_{max}	p_{FWE}	Voxels in cluster	p_{FWE}	x	y	z
**Cortical Regions**								
Sensorimotor Cortex	L	10.59	0.001	149[a]	<0.001	-52	-22	42
	L	10.47	0.001	486[b]	<0.001	-36	-20	62
	L	9.80	0.002	44[c]	<0.001	-54	4	36
	L	9.31	0.004	- part of Cluster *b* -		-36	-22	52
	L	8.79	0.007	- part of Cluster *b* -		-36	-10	54
	L	8.70	0.008	22	0.001	-6	-12	58
	L	8.23	0.014	25	<0.001	-12	-2	58
	L	8.10	0.016	44[d]	<0.001	-6	4	46
	L	8.00	0.019	14	0.002	-32	-38	50
Visual Cortex	L	10.19	0.001	87	<0.001	-50	-72	-6
	R	9.71	0.002	98[e]	<0.001	40	-66	-10
	L	9.43	0.003	45	<0.001	-28	-86	12
	L	8.89	0.006	63	<0.001	-22	-78	-12
	R	8.41	0.011	- part of Cluster *e* -		46	-58	0
	R	7.81	0.024	17	0.001	32	-88	20
Insular Cortex	R	9.32	0.004	93[f]	<0.001	36	10	4
Broca's Area	L	8.76	0.007	- part of Cluster *c* -		-56	8	28
	R	7.45	0.039	- part of Cluster *e* -		44	14	10
Inferior parietal lobule	L	8.70	0.008	- part of Cluster *a* -		-56	-20	26
Operculum	R	8.21	0.014	- part of Cluster *f* -		44	4	0
Cingulate Gyrus	L	8.06	0.017	- part of Cluster *d* -		-6	2	38
**Cerebellar Regions**								
CB V	R	9.36	0.003	69	<0.001	16	-50	-24
CB VIIIa	R	8.31	0.012	15	0.001	20	-64	-48
CB VI	L	8.14	0.016	17	0.001	-28	-60	-20
**Subcortical Regions**								
Putamen	L	11.49	<0.001	255[g]	<0.001	-28	-18	8
Pallidum	L	8.87	0.006	- part of Cluster *g* -		-22	-6	-6

Superscripts on the cluster volume, when present, refer to clusters with multiple peaks. Coordinates are expressed in MNI space.

Table S7. Background activation for ECU in the whole brain – alternative GLM analysis

**Region**		**Peak-Level**		**Cluster-Level**		**Peak coordinate** [mm]		
Area	Laterality	t_{max}	p_{FWE}	Voxels in cluster	p_{FWE}	x	y	z
**Cortical Regions**								
Sensorimotor Cortex	L	16.67	0.000	2066[a]	0.000	-8	4	36
	L	15.13	0.000	- part of Cluster *a* -		-32	-18	64
	L	12.53	0.000	- part of Cluster *a* -		-34	-20	46
	L	8.38	0.010	15	0.002	-8	-24	46
	R	7.84	0.020	18	0.002	34	-6	52
Insular Cortex	L	12.07	0.000	- part of Cluster *d* -		-36	4	0
	R	11.96	0.000	- part of Cluster *e* -		44	2	0
Visual Cortex	R	11.56	0.000	165	0.000	30	-80	18
	R	10.85	0.001	255	0.000	38	-66	-14
	R	9.89	0.002	23	0.001	16	-98	6
	L	8.08	0.015	66[b]	0.000	-30	-80	-14
	L	7.87	0.019	16	0.002	-26	-88	12
	L	7.84	0.020	- part of Cluster *b* -		-38	-78	-12
Inferior parietal lobule	L	9.15	0.004	48[c]	0.000	-58	-20	26
	L	8.59	0.008	31	0.000	-50	-30	24
	R	8.58	0.008	31	0.000	66	-18	26
	L	7.34	0.040	- part of Cluster *c* -		-58	-30	26
Superior parietal lobule	L	8.98	0.005	32	0.000	-28	-48	56
Broca's Area	L	8.92	0.005	14	0.003	-52	2	34
**Cerebellar Regions**								
CB V	R	7.78	0.022	30	0.000	18	-50	-26
CB VI	L	7.61	0.027	10	0.005	-34	-52	-20
**Subcortical Regions**								
Putamen	L	14.94	0.000	1439[d]	0.000	-8	-10	0
	R	12.58	0.000	1104[e]	0.000	30	-4	10
	L	12.16	0.000	- part of Cluster *d* -		-32	-12	-2
	R	11.90	0.000	- part of Cluster *e* -		26	-2	0

Superscripts on the cluster volume, when present, refer to clusters with multiple peaks. Coordinates are expressed in MNI space.

3 Supplementary Figures and Tables

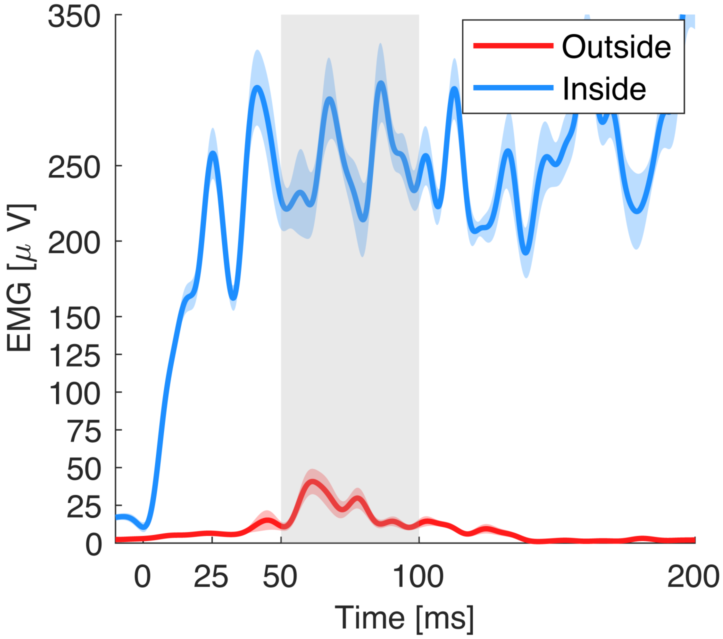

Figure S6. Stretch-evoked EMG signal measured using off-the-shelf electrodes, amplifier, and signal processing pipeline in response to a 125 deg/s RaH perturbation for the Flexor Carpi Radialis (FCR) for a representative subject.

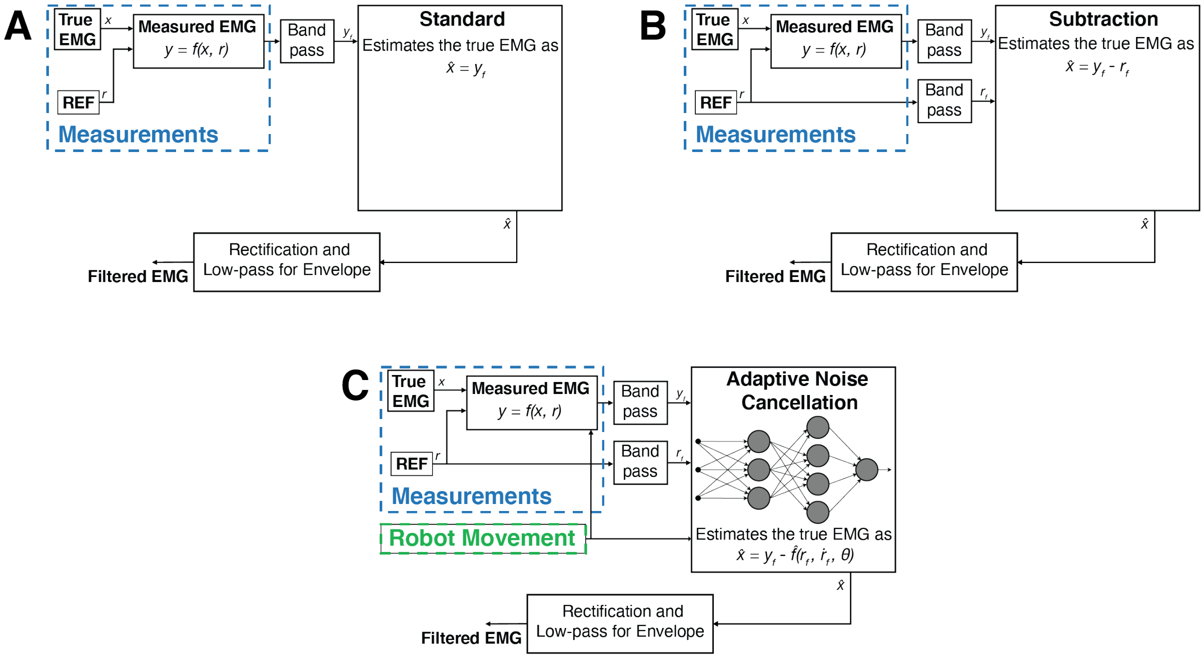

Figure S7. EMG processing schemes based on Standard (A), Subtraction (B), Adaptive Noise Cancellation (C) processing pipelines.

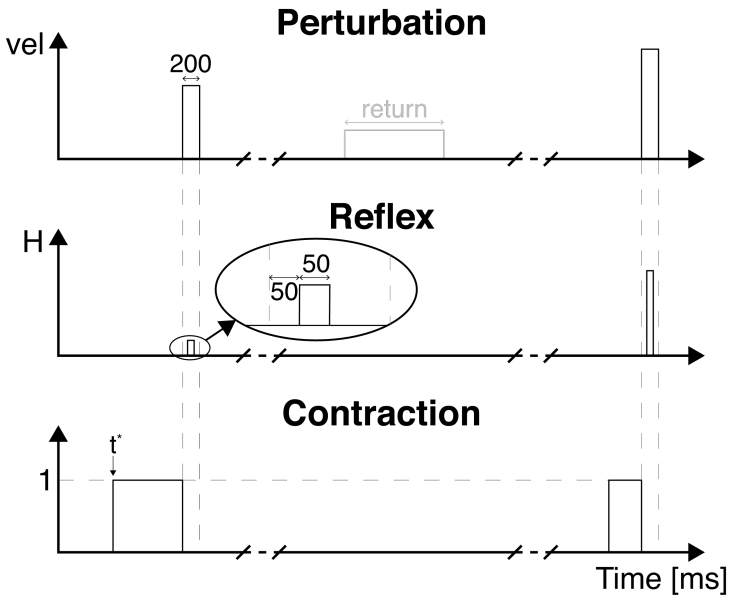

Figure S8. Graphical representation of the regressors used in the statistical analysis of the fMRI data. The value t* in the contraction regressor represents the time at which the pre-perturbation torque increased above 25% of the desired background torque.

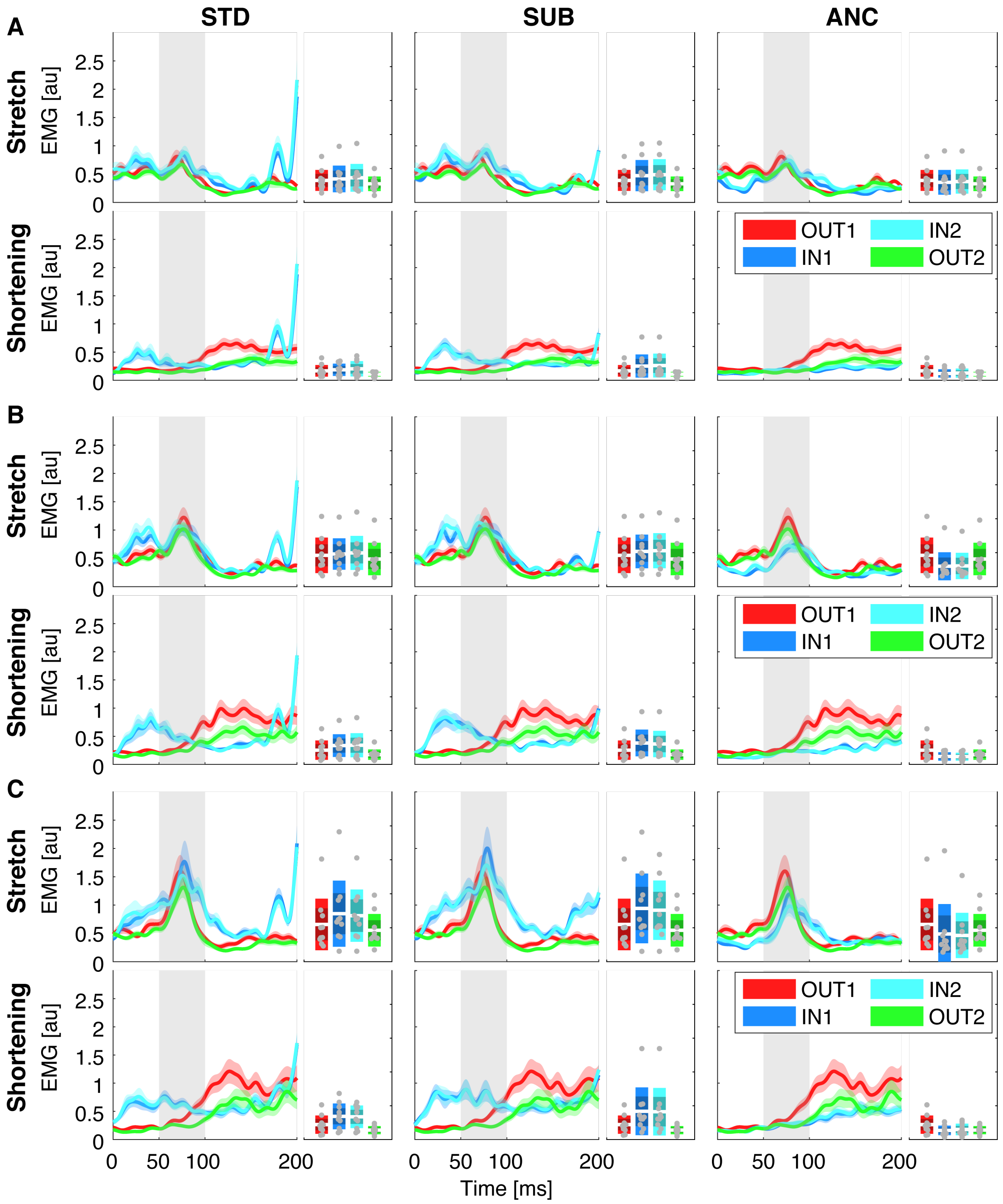

Figure S9. Group-average of the stretch-evoked EMG signal measured using different processing pipelines in response to a (**A**) 50 deg/s, (**B**) 125 deg/s, and (**C**) 200 deg/s RaH perturbation for ECU undergoing stretch or shortening for experiment 1. Plots are obtained with a combination of the novel data collection hardware with a standard signal processing pipeline (left), a time-domain subtraction of measured and reference signals (center), and the novel adaptive noise cancellation pipeline (right). In all graph the thick line represents the mean and the shaded area the 95% confidence interval. To facilitate interpretation on the graphs a gray shaded area has been included in each graph in the time interval where a LLR is expected.

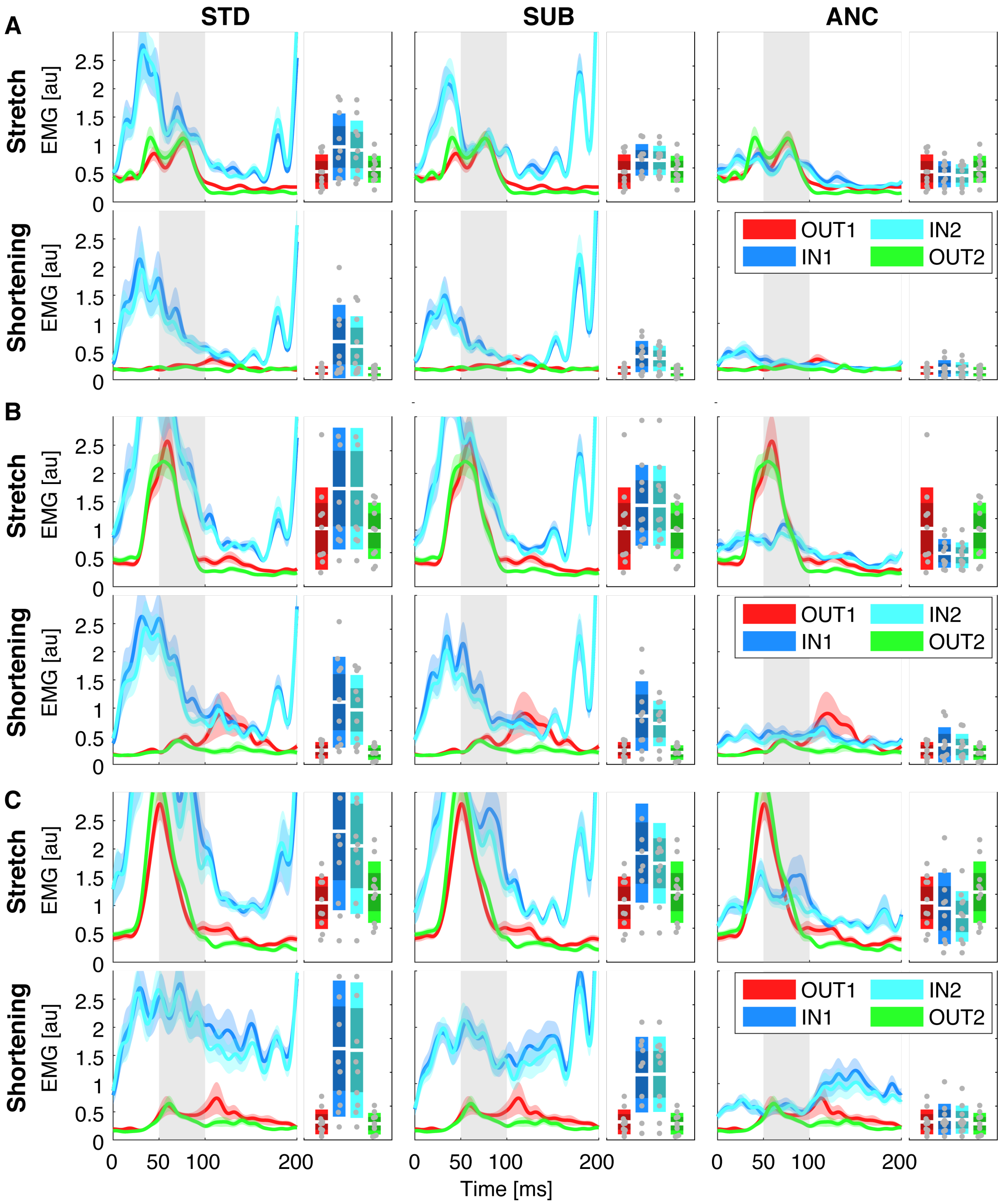

Figure S10. Group-average of the stretch-evoked EMG signal measured using different processing pipelines in response to a (**A**) 50 deg/s, (**B**) 125 deg/s, and (**C**) 200 deg/s RaH perturbation for FCR undergoing stretch or shortening for experiment 1. Plots are obtained with a combination of the novel data collection hardware with a standard signal processing pipeline (left), a time-domain subtraction of measured and reference signals (center), and the novel adaptive noise cancellation pipeline (right). In all graph the thick line represents the mean and the shaded area the 95% confidence interval. To facilitate interpretation on the graphs a gray shaded area has been included in each graph in the time interval where a LLR is expected.

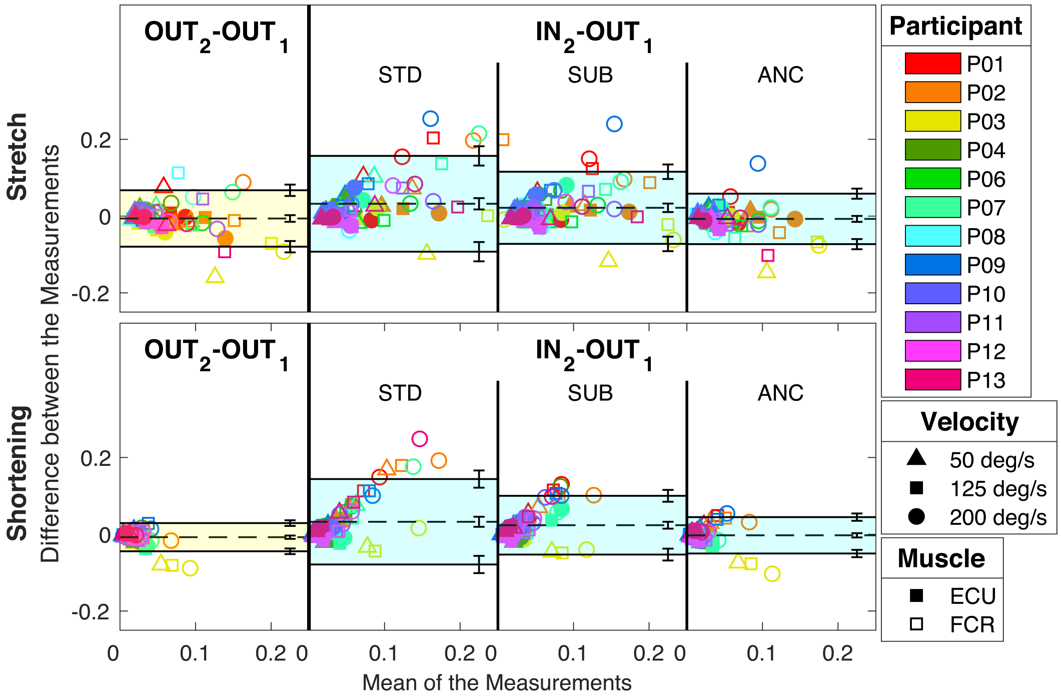

Figure S11. Bland-Altman plots for the comparisons OUT_2 vs. OUT_1 (left) and IN_2 vs. OUT_1 (right) when different filtering pipelines are considered. Datapoints in each of the plots model participants with colors, perturbation velocities with shapes, and muscles from which the EMG is recorded with fillings. The shaded area represents the interval between the negative and positive limits of agreement, while the dashed line represent the bias. Error bars on the side of each plot represent the 95% confidence intervals by which each of the parameter is estimated. Top and bottom plots distinguish the muscle activity measured in response of the two stimulus directions, respectively stretch on top and shortening on the bottom.

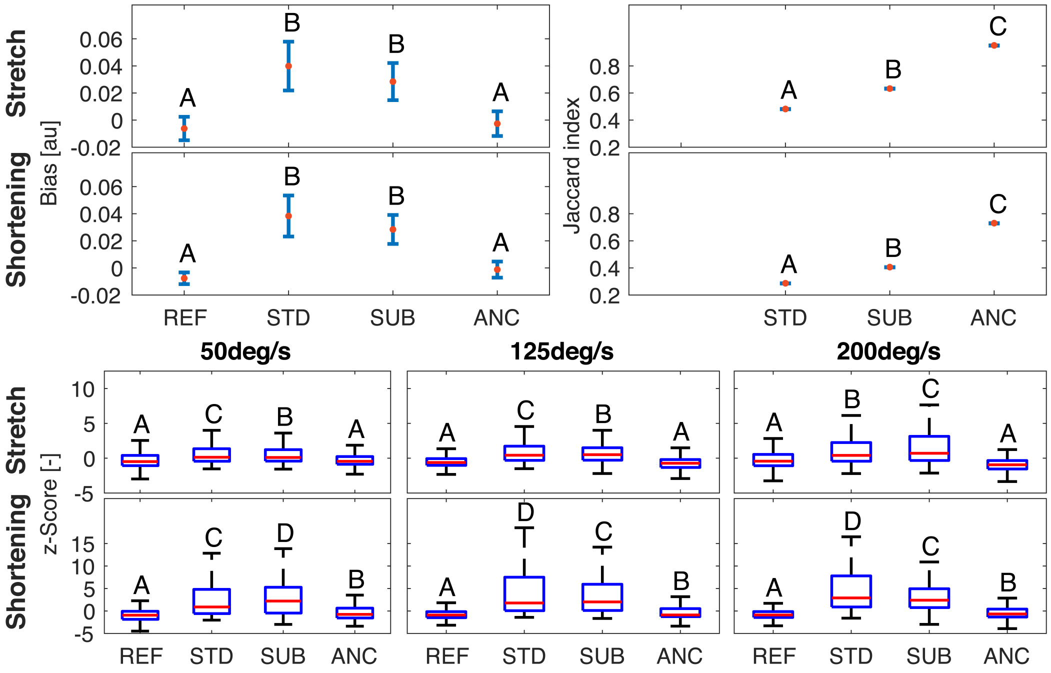

Figure S12. Mean and 95% confidence intervals for the bias (**A**), and the Jaccard index (**B**) obtained for the different filtering pipelines. Test-retest parameters measured for the two stimulus directions are reported in different rows. Letters indicate rankings of the test-retest parameters. Bars that do not share a letter have a statistically different mean. (**C**) Box plots representing the distributions of standardized *z*-scores measured for different combinations of perturbation velocity and filtering pipeline. Distributions obtained for the two stimulus directions are reported in different rows. Letters indicate rankings of the variance of each distribution; boxes that do not share a letter have a statistically different variance.

Table S8. Results of the Bland-Altman analysis for session IN_1

	**OUT_2-OUT_1**		**IN_1-OUT_1** STD		SUB		ANC	
	Stretch	Shortening	Stretch	Shortening	Stretch	Shortening	Stretch	Shortening
Bias	-0.007	-0.007	0.032	0.030	0.023	0.021	-0.001	0.002
95% CI	[-0.015, 0.001]	[-0.012, 0.003]	[0.017, 0.047]	[0.017 0.043]	[0.010, 0.035]	[0.012, 0.030]	[-0.009, 0.007]	[-0.007, 0.003]
LoA-	-0.070	-0.043	-0.096	-0.080	-0.082	-0.056	-0.072	-0.046
95% CI	[-0.082, -0.057]	[-0.050, -0.036]	[-0.123, -0.071]	[-0.102, -0.058]	[-0.103, -0.061]	[-0.072, -0.040]	[-0.087, -0.058]	[-0.055, -0.037]
LoA+	0.056	0.028	0.161	0.140	0.128	0.099	0.070	0.042
95% CI	[0.043, 0.068]	[0.021, 0.035]	[0.135, 0.187]	[0.118, 0.162]	[0.107, 0.149]	[0.084, 0.115]	[0.056, 0.085]	[0.033, 0.051]
Jaccard			0.489	0.324	0.593	0.457	0.843	0.783
95% CI			[0.4890 0.4896]	[0.3241, 03245]	[0.5930, 0.5937]	[0.4562, 0.4567]	[0.8424, 0.8432]	[0.7828 0.7836]

Table S9. Standard deviation for the set of normalized scores obtained for session IN_1

	vel [deg/s]	REF	STD	SUB	ANC
Stretch	50	0.896	1.353	1.256	0.905
	125	0.858	1.420	1.285	0.933
	200	1.175	2.287	2.118	1.021
Shortening	50	1.209	3.644	3.376	1.161
	125	1.051	4.980	3.789	1.434
	200	1.143	4.020	3.587	1.339

Table S10. Results of the Bland-Altman analysis for session IN_2

	**OUT_2-OUT_1**		**IN_2-OUT_1**					
			STD		SUB		ANC	
	Stretch	Shortening	Stretch	Shortening	Stretch	Shortening	Stretch	Shortening
Bias	-0.006	-0.008	0.032	0.033	0.022	0.024	-0.007	0.003
95% CI	[-0.015, 0.002]	[-0.012, 0.003]	[0.017, 0.047]	[0.019 0.047]	[0.011, 0.033]	[0.015, 0.033]	[-0.015, 0.001]	[-0.008, 0.003]
LoA-	-0.079	-0.044	-0.093	-0.079	-0.072	-0.053	-0.073	-0.050
95% CI	[-0.094, -0.064]	[-0.050, -0.037]	[-0.118, -0.068]	[-0.101, -0.056]	[-0.091, -0.053]	[-0.068, -0.037]	[-0.086, -0.060]	[-0.059, -0.040]
LoA+	0.067	0.029	0.157	0.144	0.116	0.100	0.058	0.044
95% CI	[0.052, 0.082]	[0.022, 0.037]	[0.132, 0.182]	[0.121, 0.166]	[0.097, 0.135]	[0.085, 0.116]	[0.045, 0.072]	[0.035, 0.054]
Jaccard			0.589	0.330	0.715	0.480	0.895	0.775
95% CI			[0.5875 0.5901]	[0.3290, 03311]	[0.7136, 0.7163]	[0.4786, 0.4810]	[0.8933, 0.8959]	[0.7740 0.7766]

Table S11. Standard deviation for the set of normalized scores obtained for session IN_2

	vel [deg/s]	REF	STD	SUB	ANC
Stretch	50	1.042	1.386	1.326	0.945
	125	0.835	1.476	1.375	0.910
	200	1.181	2.158	2.230	0.993
Shortening	50	1.299	4.173	3.832	1.562
	125	1.117	5.732	3.892	1.411
	200	1.131	4.664	3.722	1.469

Table S12. Test-retest reliability of fMRI data

H_{FCR}

		Cortical ROIs									
		M1		PM		S1		SPL		IPS	
		R	**L**	R	**L**	R	**L**	R	**L**	R	**L**
ICC	medICC	0.57 (0.01)	0.57 (0.01)	0.54 (0.01)	0.56 (0.01)	0.55 (0.00)	0.71 (0.01)	0.63 (0.00)	0.55 (0.00)	0.56 (0.03)	0.39 (0.02)
	maxICC	0.88	0.86	0.83	0.88	0.87	0.94	0.9	0.87	0.72	0.74
	ICCv	0.50 (0.01)	0.69 (0.01)	0.59 (0.01)	0.74 (0.01)	0.57 (0.01)	0.76 (0.01)	0.53 (0.02)	0.55 (0.01)	0.48 (0.02)	0.48 (0.02)
	Sg	0	0.71	0.06	0.6	0	0.61	0.1	0.24	1	0.58
	Ss	0.19 (0.01)	0.44 (0.02)	0.35 (0.02)	0.43 (0.02)	0.28 (0.01)	0.64 (0.01)	0.32 (0.02)	0.26 (0.02)	0.33 (0.03)	0.23 (0.02)

		Cortical ROIs		Subcortical ROIs					Whole Brain
		Ce		Th		Pt		Bs	
		**R**	L	R	**L**	R	**L**		
ICC	medICC	0.61 (0.00)	0.58 (0.00)	0.37 (0.01)	0.43 (0.00)	0.43 (0.01)	0.49 (0.01)	0.28 (0.01)	0.52 (0.00)
	maxICC	0.9	0.96	0.81	0.72	0.76	0.81	0.98	1
	ICCv	0.61 (0.01)	0.60 (0.01)	0.44 (0.01)	0.38 (0.01)	0.32 (0.01)	0.32 (0.01)	0.31 (0.01)	0.62 (0.01)
	Sg	0.86	0.84	0.4	0.52	0.45	0.57	0.06	0.5
	Ss	0.42 (0.02)	0.46 (0.01)	0.13 (0.01)	0.13 (0.01)	0.06 (0.01)	0.14 (0.01)	0.06 (0.01)	0.44 (0.01)

H_{ECU}

		Cortical ROIs									
		M1		PM		S1		SPL		IPS	
		R	**L**	R	**L**	R	**L**	R	**L**	R	**L**
ICC	medICC	0.51 (0.01)	0.57 (0.00)	0.37 (0.00)	0.47 (0.00)	0.49 (0.00)	0.58 (0.01)	0.58 (0.00)	0.54 (0.00)	0.40 (0.01)	0.39 (0.01)
	maxICC	0.73	0.85	0.74	0.74	0.79	0.84	0.82	0.77	0.52	0.58
	ICCv	0.48 (0.01)	0.69 (0.01)	0.56 (0.01)	0.72 (0.01)	0.65 (0.01)	0.75 (0.01)	0.61 (0.01)	0.64 (0.01)	0.47 (0.02)	0.41 (0.02)
	Sg	0	0.45	0.14	0.23	0.12	0.58	0.01	0.08	NaN	0.18
	Ss	0.16 (0.01)	0.43 (0.01)	0.26 (0.01)	0.46 (0.02)	0.27 (0.01)	0.59 (0.02)	0.26 (0.02)	0.37 (0.02)	0.10 (0.01)	0.17 (0.02)

		Cortical ROIs		Subcortical ROIs					Whole Brain
		Ce		Th		Pt		Bs	
		**R**	L	R	**L**	R	**L**		
ICC	medICC	0.50 (0.00)	0.34 (0.00)	0.39 (0.01)	0.37 (0.00)	0.55 (0.01)	0.54 (0.01)	0.24 (0.00)	0.48 (0.00)
	maxICC	0.88	0.95	0.79	0.72	0.81	0.74	0.94	1
	ICCv	0.58 (0.01)	0.53 (0.02)	0.46 (0.01)	0.41 (0.02)	0.30 (0.02)	0.34 (0.01)	0.29 (0.01)	0.62 (0.01)
	Sg	0.75	0.74	0.74	0.6	0.14	0.41	0.12	0.41
	Ss	0.39 (0.02)	0.30 (0.01)	0.13 (0.01)	0.12 (0.01)	0.18 (0.02)	0.14 (0.01)	0.06 (0.01)	0.41 (0.01)

Table S13. Number of brainstem voxels with statistically-significant activation in the participant-specific analysis ($p<0.05$)

subj ID	FCR	ECU
S04	1	10
S06	7	2
S07	6	0
S08	7	4
S09	0	1
S10	7	3
S11	3	1
S12	5	7
S14	11	4
S18	8	0
S19	4	4
S20	7	6
S21	3	1
S22	0	0
S23	4	1
S24	0	3
S25	1	0
S27	4	3

The table reports the number of voxels included in the ROI identified by the group-level analysis that showed statistically significant activity in the participant-specific analysis. The number of voxels within the ROI is 33 for FCR and 19 for ECU.

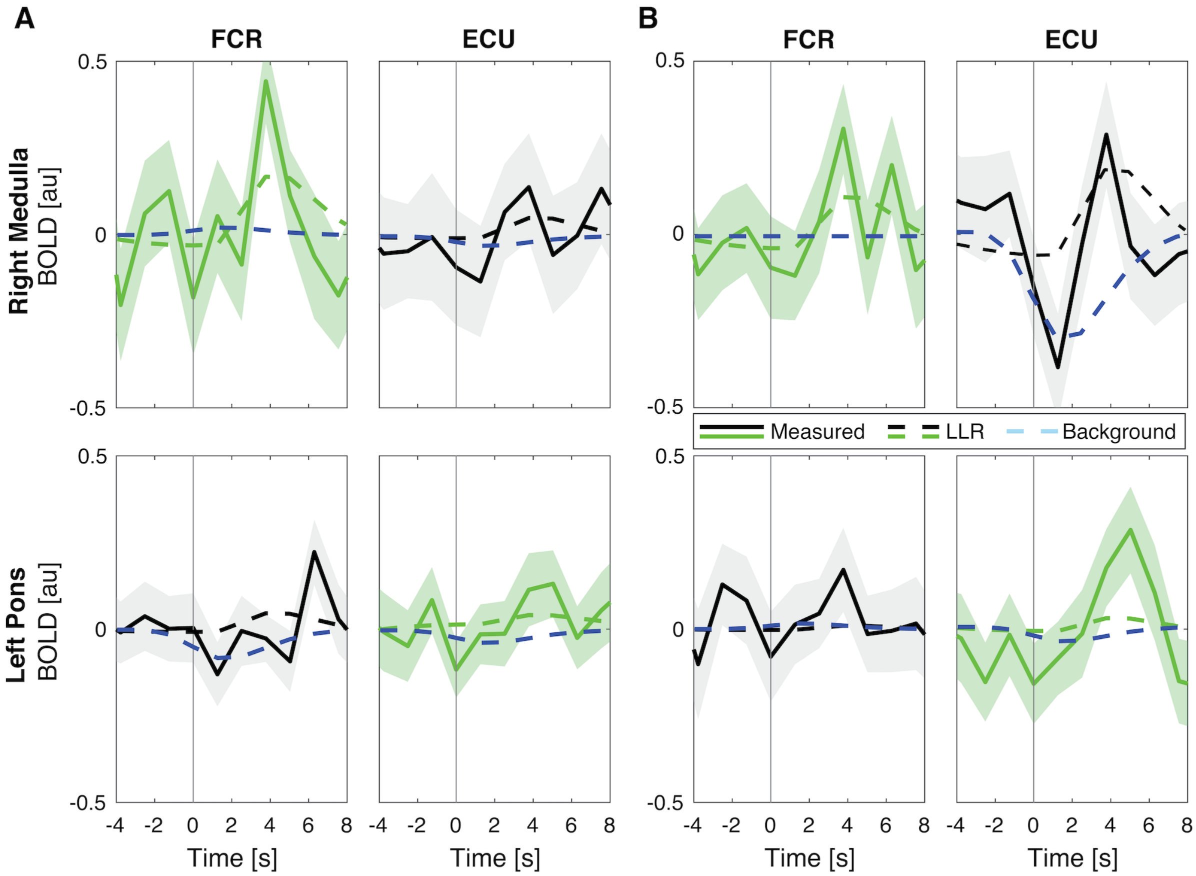

Figure S13. Average BOLD signal measured in response to muscle stretch in the selected voxels, for the selected participants. Specifically, **A** include the data that refer to the subjects that showed the greatest difference between the statistical scores specific to the FCR and ECU regressors, while **B** include the data that refer to those subject that showed similar t score for the two regressors (Fig. 6). In all graphs, the solid line shows the average residual BOLD response after variance associated to nuisance regressors is removed, while the shaded area represents the 95% confidence interval of the mean response. Dashed lines show the average modeled response respectively for the LLR-specific regressors (green or black line), and for the background-specific regressor (light blue line). Modeled response for the LLR-specific regressor was scaled based on the appropriate β coefficient estimated by the GLM model, while modeled background response, which is not included in the primary GLM, was arbitrarily scaled to match the same amplitude of the LLR-specific regressor. Measured and LLR-specific signals are then color-coded to distinguish the response that is significant at the group level for that specific muscle (green), from the one that is not significant (black).

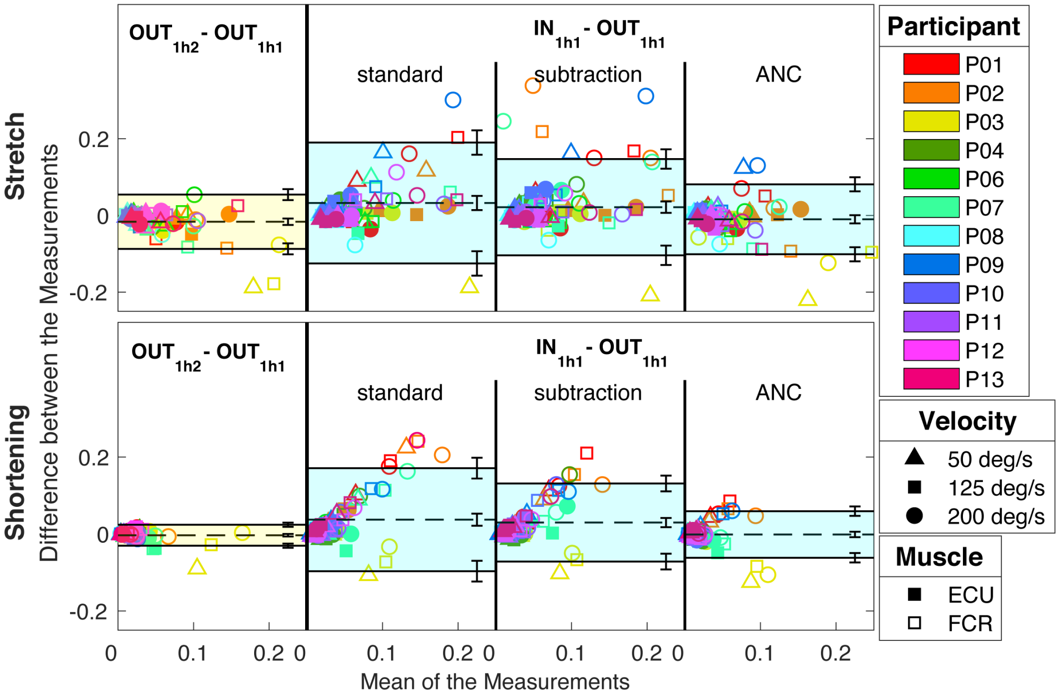

Figure S14. Bland-Altman plots for the comparisons OUT_{1h2} vs. OUT_{1h1} (left) and IN_{1h1} vs. OUT_{1h1} (right) when different filtering pipelines are considered. For both comparisons, subscripts 'h1' and 'h2' indicate the first half and the second half, respectively, of the perturbations recorded during the session. Data points in each of the plots indicate participants with colors, perturbation velocities with shapes, and muscles from which the EMG is recorded with fillings. The shaded area represents the interval between the negative and positive limits of agreement, while the dashed line represent the bias. Error bars on the side of each plot represent the 95% confidence intervals by which each of the parameter is estimated. Top and bottom plots distinguish the muscle activity measured in response of the two stimulus directions, respectively stretch on top and shortening on the bottom.

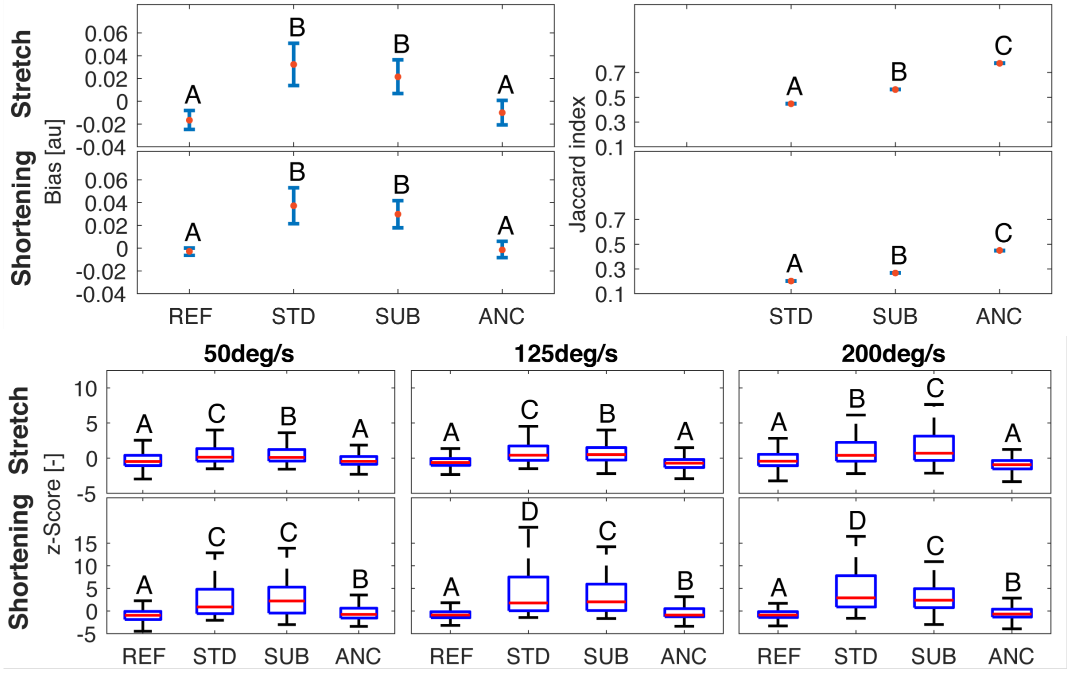

Figure S15. Mean and 95% confidence intervals for the bias (**A**), and the Jaccard index (**B**) obtained for the different filtering pipelines. Here the REF condition corresponds to comparisons between OUT_{1h2} vs. OUT_{1h1}sessions, and the test conditions are calculated in the first half of contractions during MRI. Test-retest parameters measured for the two stimulus directions are reported in different rows. Letters indicate rankings of the test-retest parameters. Bars that do not share a letter have a statistically different mean. (**C**) Box plots representing the distributions of standardized z-scores measured for different combinations of perturbation velocity and filtering pipeline. Distributions obtained for the two stimulus directions are reported in different rows. Letters indicate rankings of the variance of each distribution; boxes that do not share a letter have a statistically different variance.

References

1. Farrens, A. J. & Sergi, F. Identifying the neural representation of fast and slow states in force field adaptation via fMRI. *IEEE Int. Conf. on Rehabil. Robotics* **2019-June**, 1007–1012, DOI: 10.1109/ICORR.2019.8779512 (2019).

2. Kim, J. H. *et al.* Characterization of BOLD hemodynamic response function in subcortical and cortical regions of human brain at 3T and 9.4 T. *24th Annu. Meet. Organ. for Hum. Brain Mapp.* (2019).
